# Efficacy of Seren@ctif, a Computer-Based Stress Management Program for Patients With Adjustment Disorder With Anxiety: Protocol for a Controlled Trial

**DOI:** 10.2196/resprot.7976

**Published:** 2017-10-02

**Authors:** Dominique Servant, Anne-Claire Leterme, Olivia Barasino, Laure Rougegrez, Alain Duhamel, Guillaume Vaiva

**Affiliations:** ^1^ Stress and Anxiety Unit Department of Psychiatry University Hospital Lille France; ^2^ Unité Mixte de Recherche 9193 Sciences Cognitives et Sciences Affectives Centre National de la Recherche Scientifique Department of Psychiatry University Hospital Lille Lille France; ^3^ Centre d'Etudes et de Recherche en Informatique Médicale Department of Biostatistics University of Lille Lille France

**Keywords:** computer-assisted therapy, eHealth, cognitive therapy, behavior therapy, psychological stress, adjustment disorders, randomized controlled trial

## Abstract

**Background:**

Adjustment disorder with anxiety (ADA) is the most frequent and best characterized stress-related psychiatric disorder. The rationale for prescription of benzodiazepine monotherapy is a public health issue. Cognitive behavioral stress management programs have been studied in many countries. Several reports have shown beyond reasonable doubt their efficiency at reducing perceived stress and anxiety symptoms and improving patient quality of life. Considering the number of people who could benefit from such programs but are unable to access them, self-help programs have been offered. First presented as books, these programs became enriched with computer-based and digital supports. Regrettably, programs for stress management based on cognitive behavioral therapy (CBT), both face-to-face and digital support, have been only minimally evaluated in France. To our knowledge, the Seren@ctif program is the first French language self-help program for stress management using digital supports.

**Objective:**

The aim of this study is to assess the effectiveness of a 5-week standardized stress management program for reducing anxiety conducted via eLearning (iCBT) or through face-to-face interviews (CBT) with patients suffering from ADA compared with a wait list control group (WLC). These patients seek treatment in a psychiatric unit for anxiety disorders at a university hospital. The primary outcome is change in the State Trait Anxiety Inventory scale trait subscale (STAI-T) between baseline and 2-month visit.

**Methods:**

This is a multicenter, prospective, open label, randomized controlled study in 3 parallel groups with balanced randomization (1:1:1): computer-based stress management with minimal contact (not fully automated) (group 1), stress management with face-to-face interviews (group 2), and a WLC group that receives usual health care from a general practitioner (group 3). Programs are based on standard CBT principles and include 5 modules in 5 weekly sessions that include the following topics: stress and stress reaction and assessment; deep respiration and relaxation techniques; cognitive restructuring, mindfulness, and acceptance; behavioral skills as problem solving; and time management, healthy behaviors, and emotion regulation. In the Internet-based group, patients have minimal contact with a medical professional before and after every session. In the first session, a flash memory drive is supplied containing videos, audio files, a self-help book portfolio in the form of an eGuide, and log books providing the exercises to be completed between 2 sessions. The patient is encouraged to practice a 20-minute daily exercise 5 or 6 times per week. In the face-to-face group, patients receive the same program from a therapist with 5 weekly sessions without digital support. Interviews and self-assessments were collected face-to-face with the investigator.

**Results:**

The feasibility of this program is being tested, and results show good accessibility in terms of acceptance, understanding, and treatment credibility. Results are expected in 2018.

**Conclusions:**

To our knowledge, this is the first French study to examine the effectiveness of a computer-based stress management program for patients with ADA. The Seren@ctif program may be useful within the framework of a psychoeducative approach. It could also be advised for people suffering from other diseases related to stress and for people with a clinical level of perceived stress.

**Trial Registration:**

Clinicaltrials.gov NCT02621775; https://clinicaltrials.gov/ct2/show/NCT02621775 (Archived by WebCite at http://www.webcitation.org/6tQrkPs1u)

## Introduction

### Background

Numerous studies have shown that exposure to a stressor increases the risk of psychiatric symptoms and disorders, especially anxiety. When anxiety symptoms are at a higher level than a normal reaction to a stressful event, we consider the possibility of a diagnosis of adjustment disorders, which are classified as stress-related disorders. They are responsible for significant direct and indirect costs from treatment, work stoppages, and loss of productivity [1].

### Adjustment Disorder With Anxiety

#### Definition

Adjustment disorder with anxiety (ADA) is the most frequent and best characterized stress-related psychiatric disorder [2]. According to the *Diagnostic and Statistical Manual of Mental Disorders, Fifth Edition* (DSM-5), a diagnosis of ADA is appropriate with the finding of anxiety occurring within 3 months of a psychosocial stressor or life event (eg, divorce, job loss, serious physical illness) [3] with symptoms generally abating by 6 months after the event. Considered a common disorder ranging from mild to moderate intensity, ADA is a true diagnostic entity. A general medical study showed that the level of anxiety is comparable to that of other anxiety disorders such as generalized anxiety disorder [4].

#### Current Recommendations for Adjustment Disorder With Anxiety

Patients are generally not treated with a tailored approach but with medication, mainly benzodiazepine monotherapy. Benzodiazepine misuse is a public health problem. Although stress management is considered the most appropriate psychological treatment for ADA, the evidence base for this approach is limited [5] **.** However, several studies compared cognitive behavioral therapy (CBT) with other treatments for generalized anxiety disorders and found greater improvements in stress and anxiety symptoms from CBT. In a study comparing CBT with relaxation, a posttreatment progression for State-Trait Anxiety Inventory scale (STAI-T trait subscale) scores from 53.04 to 46 and for Penn State Worry Questionnaire (PSWQ) scores from 61 to 51.13 were shown for the group that received CBT [6]. A study comparing psychodynamic psychotherapy with CBT found a greater improvement in the symptoms of stress and anxiety for the CBT intervention (a posttreatment progression of STAI-T trait subscale scores from 58.83 to 43.41 and of PSWQ scores from 63.48 to 49.86 in this group) [7]. In addition, eMental health options are considered uniquely suited for offering early intervention after patients experience stressful life events that can potentially trigger adjustment disorders [8].

#### Development of Preventive and Curative Measures for Adjustment Disorder With Anxiety

It seems legitimate, given the prevalence and the human, economic, and social costs of this pathology linked to stress, to develop preventive and curative measures for ADA. It is not always possible to intervene upstream to reduce the exposure to stress and prevent the occurrence of ADA (primary prevention). Secondary and tertiary prevention measures are therefore useful to limit their consequences.

### Stress and Anxiety Management

Stress management is a set of educational and psychotherapeutic measures that combines several cognitive behavioral techniques. The aim is to allow the patient to reach a satisfactory level of emotional and cognitive control and cope with stressors in order to reduce the negative consequences. The common practice is to offer group sessions or individual interviews in the form of a structured module limited in time [9].

### Cognitive Behavioral Therapy

CBT has been shown to be an effective treatment for reducing anxiety symptoms in various somatic pathologies such as cardiovascular disease [10,11], diabetes [12], chronic fatigue syndrome [13], and breast cancer [14] and in subjects with high levels of perceived stress or anxiety or burnout, particularly in the workplace [15]. A meta-analysis by Richardson and Rothstein [16] showed a moderate effect size (*d*=0.53) of stress management programs in the context of work, which is considered an average effect size according to the Cohen criteria [17]. For structured programs based on CBT, the effect is clearly greater (*d*=1) [9].

### Self-Help Therapy

Given that many patients do not have any access to stress and anxiety management programs, therapeutic education modules based on guides and self-help books have been offered and have shown positive results compared with classical CBT programs and control groups [18,19].

### Digital Stress Management

In recent years, the development of new technologies has enriched self-help programs by integrating new tools (CD-ROM, flash memory drive, or directly accessible on the Internet), allowing better interactivity and use other than contact with the therapist for patients [20].

These computer-assisted programs are intended to limit the amount of contact time with a mental health professional [21-23]. They have been evaluated in many countries in general, student, and corporate populations [24-36]. Unfortunately, computer-assisted programs have not been evaluated in France.

Recently, a meta-analysis evaluating the effect sizes of 26 computer- and Internet-based interventions was conducted on psychological stress and found significant results in terms of reduction of symptoms of stress (Cohen *d*=0.43, 95% CI 0.31-0.54), depression (Cohen *d*=0.34, 95% CI 0.21-0.48), and anxiety (Cohen *d*=0.32, 95% CI 0.17-0.47) compared to other types of interventions. These results provide evidence that Web- and computer-based stress management interventions can be effective and have the potential to reduce stress-related mental health problems on a large scale [37].

### Development of the Seren@ctif Program

It was in this context that Seren@ctif, a computer-based self-help management program, was developed at Lille University Hospital. It is the first French CBT program using digital support (iCBT). Seren@ctif is a neologism formed from 2 French words: seren for *serenité*, which means serenity, and actif, which means active and refers to self-help. The words are joined with an “at” sign to indicate the use of a digital format.

A pilot study was carried out between January and June 2014 to study the feasibility of this therapeutic program [38]. The results are satisfactory (average scores for satisfaction questionnaires were high, with scores ranging from 4 to 5 on a 5-point Likert scale). These results were the reason why it seemed appropriate to go further by evaluating this program in a controlled manner.

### Aims of the Study

The primary objective of this study is to assess the effectiveness of our stress management program conducted via e-learning (iCBT) or through face-to-face sessions compared with a wait list control (WLC) to reduce the anxiety level after 2 months in patients with a diagnosis of ADA.

The primary outcome is the change in the STAI-T score between the baseline and the 2-month visits.

The secondary objectives of this study are as follows:

- Evaluate the maintenance of effectiveness of the 2 therapeutic stress management programs (iCBT and CBT) at 6 months
- Compare the change in stress, worry, anxiety, and depressive symptoms in the 2 therapeutic programs at 2 and 6 months
- Measure the impact of the 2 therapeutic programs on the consumption of tobacco, alcohol, and drugs
- Assess overall satisfaction with the 2 therapeutic programs at 2 and 6 months and with the WLC at 6 months

The secondary outcome measures include the following:

- Change in STAI-T scores at 6 months
- Hospital Anxiety Depression Scale (HADS) anxiety subscale scores at 2 and 6 months
- Worry symptoms evaluated by the PSWQ
- Stress level evaluated by the Perceived Stress Scale (PSS) and the Visual Analog Scale–stress (VAS-stress) at 2 and 6 months
- Depressive symptoms evaluated by the Beck Depression Inventory (BDI) and the HADS depression subscale at 2 and 6 months
- Overall satisfaction evaluated by the VAS-satisfaction at 2 and 6 months for the 2 therapeutic groups and at 6 months for the WLC group
- Change in consumption of tobacco, alcohol, and drugs evaluated at 2 and 6 months

### Hypotheses

We hypothesize that (1) both therapeutic programs will have a greater clinical impact on the reduction of anxiety symptoms, perceived stress, and depressive symptoms compared with a WLC in the short term (2 months), and this effect will be maintained in the long term (6 months); (2) the 2 therapeutic programs will have the same effectiveness in reducing these symptoms; (3) both therapies will be more cost effective than will WLC; (4) these 2 programs both reduce the consumption of tobacco, alcohol, and drugs compared with WLC; and (5) participants will be satisfied with the 2 therapeutic programs.

### Choice of Comparators

The comparison with the program implemented face-to-face is intended to highlight the value of eLearning, and the comparison with the control group, whose members receive general medical care, is intended for comparison with the current recommendations for therapeutic studies.

### Prospect of the Project

The future goal of this project is to enrich the program with new information and communication technologies, such as the Internet (eCBT) [39-41] and mobile (mCBT) options [42-45], and propose the program to a large population for prevention of stress-related disorders.

## Methods

### Setting and Procedure

#### Type of Study

It is a multicenter, comparative, prospective, unblinded, randomized, controlled study in 3 parallel groups. As it is not possible to mask the different treatment groups, patients were not blinded from their intervention group.

#### Ethics

The project was approved by the local ethics committee, *Comité de Protection des Personnes Nord Ouest IV* (approval number CPP 15/12), as is required for medical intervention research in France. Data processing will be carried out in accordance with the requirements of the *Commission Nationale de l’Informatique et des Libertés* reference methodology, MR 06001 (Multimedia Appendix 1).

#### Recruitment

The study is being conducted at 3 university hospitals (Lille, Amiens, Caen) in the northwest area of France as part of the university communities (*InterRégional*). Patients are referred by their general practitioner to a psychiatric consultation service for psychological treatment of anxiety symptoms in a context of recent stress. To improve recruitment, general practitioners in the 3 areas were informed of the study by a local investigator during continuing medical education, during scientific meetings, and through all types of collaborative contacts between primary care and psychiatry services. We also directly informed the general practitioners who were involved in previous research on ADA and CBT and invited them to refer patients.

#### Agreement of the Subjects

During a medical interview, participants will receive oral and written information detailing the progress of the trial and be allowed a period of reflection. Informed consent will be collected from each subject before they enter the study (Multimedia Appendix 2).

#### Criteria for Discontinuing Participation in the Study

Participants are told they are free to leave the study at any time. Participants will be released from the study by the investigator in cases of adverse events such as diagnosis of an illness requiring hospitalization or surgery, beginning a new medication, or changing medication doses. The current research does not involve any risk, with the exception of the possible negative psychological impact of completing the psychological questionnaires.

#### Eligibility

Each newly referred patient is asked to answer the optional adjustment disorder section of the Mini International Neuropsychiatric Interview (MINI) [46], French version [47], to confirm he or she meets to the ADA criteria according the DSM-5. The MINI is administered during a face-to-face interview by clinical investigators who are trained in psychiatry. See Textbox 1 for selection criteria for study subjects.

#### Randomization

Immediately after inclusion and assessment, patients are randomly allocated in a 1:1:1 ratio into 3 groups using a Web-based central randomization: patients using a digital stress management module (group 1), patients following the stress management module guided by a therapist in attendance (group 2), and patients on a waiting list and benefiting from usual care through their attending physician (group 3).

The randomization sequence is provided by an independent statistician (who does not take part in assessing the patients at any point in the study) using computer-generated random numbers with block sizes of 6 and center stratification consistent with the Consolidated Standards of Reporting Trials (CONSORT) [48]. The randomization sequence is implemented in the electronic case report form (eCRF) system to ensure a centralized, real-time randomization procedure.

A document describing the randomization procedure is kept confidential in the Clinical Investigation Centre of Lille University Hospital.

#### Assessments

Assessments are conducted for the 3 groups at baseline, 2 months, and 6 months. At baseline, participants complete the self-administered questionnaires for anxiety and depressive symptoms, and care contacts and medications are recorded. At 2 and 6 months, an evaluation of overall satisfaction as well as adverse events is additionally carried out. Self-assessments are collected in the face-to-face sessions with the investigator.

It is the same psychiatrist investigator for each site who generated the random allocation sequence, enrolled the participants, assigned the participants to the interventions, and assessed the participants, so he is not blinded to the intervention group.

**Figure 1:**
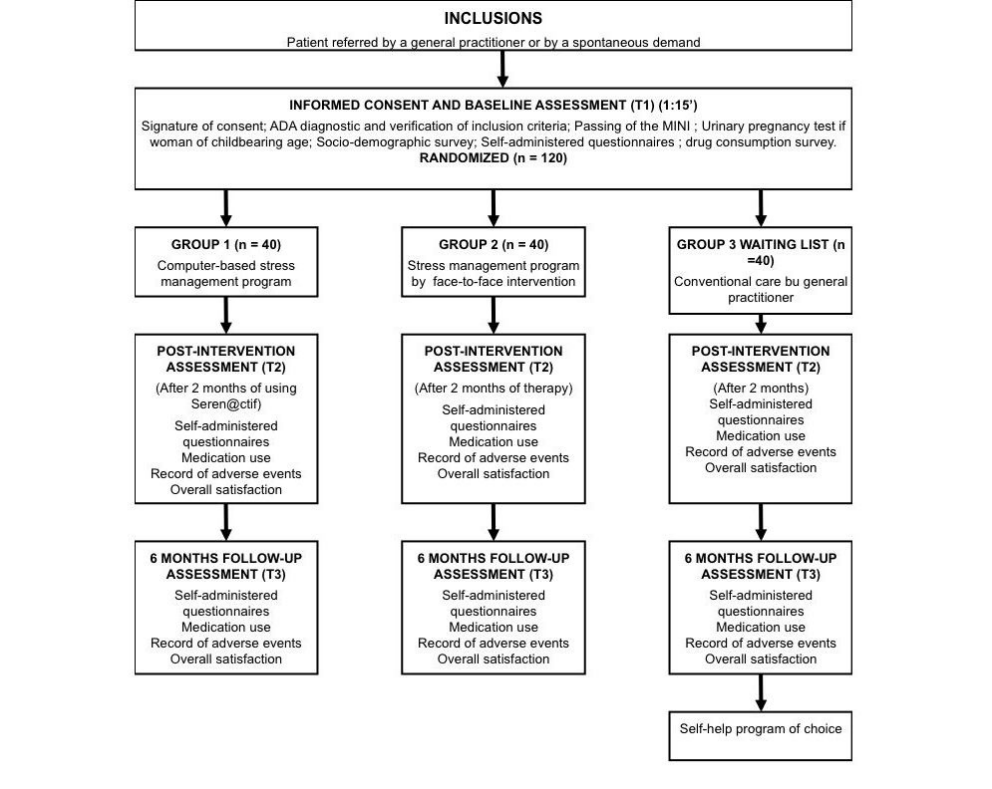
Flowchart of the study.

The flowchart in Figure 1 summarizes the experimental design.

### Interventions

The content of the 2 programs is identical; one is delivered by computer and the other face-to-face. The details of the programs are presented in Table 1.

#### Computer-Based Stress Management Program

The program includes 5 weekly sessions lasting 1 hour that patients follow from a program accessible on a computer in our unit.

Participants have minimal contact with a clinician to encourage adherence and engagement with the program. This minimal contact of 5 minutes is performed before and after every session by a trained nurse, who investigates the adverse events and drug dose changes since the last session, answers any questions, discusses the progress of the session, and possibly guides participants in the navigation of the computer program.

To avoid connection problems, a flash memory drive is supplied to the participant at the first session that contains videos, audio files, self-help books, a portfolio in the form of an eGuide, and a log book with the program of exercises to be completed between 2 successive sessions of the program. The patient is encouraged to practice 1 or several daily exercises for 20 minutes each, 5 or 6 days per week (Multimedia Appendix 3).

**Selection criteria.**

Inclusion criteria:

- Ambulatory patient (as inpatients are frequently a mixed anxiety depressive type of adjustment disorder)
- Male or female aged 18 to 60 years (we use a cutoff age of 60 years for inclusion to limit chronic somatic comorbidities)
- Diagnosis of adjustment disorder with anxiety (ADA) according to the *Diagnostic and Statistical Manual of Mental Disorders, Fifth Edition* criteria
- Currently not supported by structured psychotherapeutic treatment for ADA or any other problem
- Taking no new psychotropic drug therapy or stabilized for at least 3 months (in the latter case, the patient will be informed of the need to keep the same dosages for the duration of the study)
- Minimum score on the Hospital Anxiety and Depression scale (HADS) anxiety subscale greater than or equal to 10 and a maximum score on the HADS depression subscale of less than 10
- Access to a computer

Exclusion criteria:

- Inability to read or use a computer with support (the platform is easy-to-use and a nurse is available to guide the patient in the navigation of the computer program)
- Pregnancy (as recommended by the French ethics committee; urine pregnancy test performed on female patients)
- Not capable of consenting, not having legal protection, or being deprived of liberty
- Diagnosis of another psychiatric disorder (according to the Mini International Neuropsychiatric Interview)

**Table 1:** Seren@ctif content.

Module theme	Aims of the module	Content of the module
Introduction and education regarding stress and anxiety	Provide psychoeducation about stress and anxiety and their treatment; identify anxiety symptoms and discuss treatment goals and expectations.	Psychoeducation about stress and anxiety (the model of the cognitive emotional stress spiral from a concrete example of stressful situations in everyday life); query about anxiety symptoms, treatment goal and expectations. Daily exercise: anxiety monitoring.
Body relaxation	Introduce different relaxation techniques and their respective targets and learn to practice them.	Different relaxation techniques: deep breathing, progressive muscle relaxation, and imagery and their relevance to stressful situations. Daily exercise: podcasts.
Cognitive therapy	Introduce the concept of changing thoughts; explain common thinking errors, alternative thoughts, and coping statements; practice.	Cognitive work on automatic thoughts based on concrete situations in everyday life; rationalization of the content of thoughts through cognitive restructuring and identification of repetitive negative ruminations. Daily exercise: identify dysfunctional automatic thoughts and search for other less stressful thoughts.
Mindfulness	Understand the interest in accepting rather than repressing emotions to focus attention on the present moment; focus on sensations to relieve negative thoughts and sensations.	Direct attention to breathing and bodily sensations to overcome stressful thoughts and accept the world and its surrounding thoughts. Daily exercise: podcasts.
Exposure and developing positive attitudes	Introduce the concept of changing behavior and practice it.	Principles of problem-solving, exposition, time management, planning pleasant activities, developing empathy, better assertiveness, exercise of full consciousness by concentration on the present moment.

#### Stress Management by Face-to-Face Interviews

The program includes 5 weekly sessions lasting 45 minutes or 1 hour with trained clinical psychologists (graduate of a master’s program in cognitive and emotional therapy with a minimum of 1 year of practice in CBT and cognitive behavioral stress management). Information, exercises, and homework assignments are delivered by the therapist without self-help support. At the beginning of each session, the therapist asks the participant about adverse events and changes in drug doses since the last session.

#### Wait List Control

Control group patients received usual care consisting of contact with their general practitioner and nonspecific psychoeducation about stress and anxiety. Medication is prescribed as a stable dose during the period prior to assessment at 2 months.

### Outcome Measures

We used the French version of self–report measures for which reliability and validity have been demonstrated.

#### Anxiety

- Spielberger State Trait Anxiety Inventory (STAI)–Form Y Trait version [49]. The French adaptation by Bruchon-Schweitzer and Paulhan [50] is a 20-item questionnaire with 4 levels of ratings from 1=not at all to 4=much (total score of 20 to 80) that captures how the subjects feel generally (9 reversed items).
- Hospital Anxiety and Depression Scale (HADS). This questionnaire queries anxiety and depressive disorders using 14 items rated on a 0 to 3 scale with 7 questions relate to anxiety (total A). A score between 8 and 10 on each of the subscales is considered a risk (possible or probable) of anxiety or depressive disorders [51]. The French version is used [52].
- Penn State Worry Questionnaire (PSWQ). This is a self-assessment questionnaire consisting of 16 items measuring the general tendency to worry with answers based on a 5-point Likert scale ranging from 1=not at all characteristic to 5=extremely characteristic (scores range from 16 to 80) [53]. A French version of the PSWQ is used [54].

#### Stress

- Perceived Stress Scale (PSS) comes from the stress transactional model and contains 14 items. The total score ranges from 0 to 56, and higher scores represent higher stress levels. Two dimensions emerge from this scale: perceived threat and perceived personal effectiveness [55]. The French version is used [56].
- Visual analog scale of stress (VAS-stress) is often used to measure the intensity of various symptoms, especially pain. It was used for the first time in 1996 for a subjective assessment of stress. The simplest VAS scale is a horizontal segment whose ends are defined as the limits of the parameter to be measured, oriented from the best to the worst. A study found external validity for the French version of VAS-stress by comparing its scores with the PSS, and it has a good sensitivity/specificity ratio [57].

#### Depression

- Abbreviated Beck Depression Inventory (BDI) is a self-assessment questionnaire measuring the severity of depression with 13 items rated from 0 to 3 [58,59].
- Hospital Anxiety and Depression Scale (HADS) assesses anxiety and depressive disorders using 14 items rated 0 to 3 with 7 questions relative to depressive symptoms (total D). A score between 8 and 10 on each of the subscales is considered a risk (possible or probable) of anxiety or depressive disorders [52].

#### Interview for Diagnosis of Psychiatric Disorders

The Mini International Neuropsychiatric Interview Version 5.0.0 (MINI) is a fully structured diagnostic interview that assesses a major axis for the diagnosis of disorders [46]. Each newly referred patient was asked to answer the optional adjustment disorder section of the MINI from the French version [47]. The MINI was administered by research assistants who were trained to established reliability criteria. Any participant who met diagnostic criteria for a DSM-5 Axis I diagnosis was excluded from the study. There was no major change between DSM-IV and DSM-5 for anxiety and depressive disorders.

#### Satisfaction

The visual analog scale of satisfaction (VAS-satisfaction) measures the overall satisfaction with the program.

#### Main Criterion

The change in the STAI scale between baseline and the 2-month visit was the main criterion. We chose this criterion because it is a self-report measure, and self-report is commonly used in CBT and seems particularly indicated for Internet-based interventions [60]. In addition, the trait of anxiety is the core of a diagnosis of ADA, and the STAI-T measures the trait of anxiety, which is distinguished from a state of anxiety. The HADS scale and the Hamilton Anxiety Rating scale do not make this distinction.

#### Secondary Criteria

- Change in the STAI scale between baseline and the 6-month visit
- Changes in other validated self-report measures of anxiety and worry at 2 and 6 months (HADS anxiety subscale, PSWQ)
- Changes in validated self-report measures of stress at 2 and 6 months (VAS-stress, PSS)
- Changes in validated self-report measures of depression at 2 and 6 months (BDI, HADS depression subscale)
- Consumption of tobacco (number of cigarettes smoked per day), alcohol (number of drinks of alcohol per week—in France, the standard drink contains 10 grams of alcohol) and drug use (days per week for each product) at 2 and 6 months
- VAS-satisfaction at 2 and 6 months for the 2 therapeutic groups and at 6 months for the WLC group

#### Sample Size

In a study on the clinical and psychometric characteristics of ADA, the mean (standard deviation) of the STAI baseline score was 52.1 (SD 14.6) [4]. Two clinical studies with patients with generalized anxiety disorder, 1 comparing CBT with relaxation [61] and the other comparing CBT with brief psychodynamic psychotherapy [62], showed changes in the STAI-T scores of 7 and 16, respectively. In view of these results, we hypothesize that an average difference between each experimental arm and the control arm of 11.5 (the mean of 7 and 16). To demonstrate this difference in variation with a standard deviation of the STAI score change of 14.6 (assuming a conservative correlation coefficient of 0.5 between the baseline and 2-month STAI measures), a 2.5% type I error (using a Bonferroni correction for the 2 prespecified comparisons), and a power of 80%, it is necessary to include 32 patients per group. Although analysis of the primary outcome will be adjusted for baseline values, the sample size calculation does not take into account this adjustment to maximize the power. Finally, considering 20% ​​missing data, 120 patients (40 per group) are planned in this clinical trial.

#### Data Collection and Management

Data are recorded in an eCRF developed using Clinsight (Ennov Inc). The eCRF is used for data entry at each investigator site, and every center is responsible for patient anonymization. The eCRF was created, tested, and validated before the start of data entry. The data necessary for monitoring the primary and secondary endpoints are identified and managed at regular intervals throughout the trial. Data are monitored by the data management team of the data management department of University Hospital of Lille using predefined data management rules, and queries are automatically edited. Finally, overall automated monitoring is performed by the data manager at the end of data entry. In case of discrepancies, queries are edited to resolve the problems encountered. After validation, the database is frozen and exported for analysis.

### Statistical Analysis

#### Overview

Statistical analysis will be conducted in a blinded fashion with a blinded code for the intervention. All statistical analyses will be carried out independently in the Department of Biostatistics of the University Hospital of Lille. SAS 9.3 (SAS Institute Inc) software or later will be used. *P* values will be reported as the actual values, unless *P*<.001. No interim analyses are planned. A detailed statistical analysis plan (SAP) will be drafted and validated before the database is frozen. Patient characteristics at baseline will be described for each of the 3 arms; the quantitative variables will be described either by the mean and standard deviation in case of a Gaussian distribution or by the median and interquartile range if not. The normality of the distributions will be verified graphically by histograms and by the Shapiro-Wilk test. The qualitative variables will be described by the numbers and percentages of each category. All analyses for the primary and secondary objectives will be performed on all randomized patients in their original group of randomization according to intention-to-treat (ITT) principles. No subgroup analysis will be performed.

#### Main Objective

Comparison of the primary endpoint (2-month change in STAI scale) between each experimental group and the control group will be performed separately using an analysis of covariance with an adjustment for the STAI baseline value and the center. Since 2 comparisons will be made for the primary analysis, each of them will be performed at the 2.5% significance level (Bonferroni correction). The absolute mean differences and effect sizes (standardized mean difference) will be reported with the 95% confidence interval. In case of deviation from normality of model residuals, nonparametric analysis will be used; absolute changes between baseline and 2-month visits will be calculated and compared between the experimental arm and control arm using nonparametric analysis of covariance adjusted for baseline values [63,64]. The primary analysis will be conducted according to the ITT principle. Missing values will be handled by multiple imputation procedures. Missing data will be imputed under the missing at random assumption using a regression switching approach (chained equation with m=20 imputations) with predictive mean matching method for continuous variables and logistic regression (binary, ordinal, or polynomial) for qualitative variables [65]. The imputation procedure will be performed using the main baseline characteristics, outcome, and variable group of randomization, and multiple imputed data sets will be combined using Rubin’s rules [66,67]. Sensitivity analyses will first be conducted using all available STAI measurements (complete cases analysis) and second in patients without major deviation from protocol (per protocol analysis); major deviations will be specified in the SAP.

#### Secondary Objectives

For the secondary endpoints of the HADS anxiety subscale, PSWQ, VAS-stress, PSS, BDI, and HADS depression subscale scores, comparisons of the changes between the baseline and the 2-month visit between each experimental group and the control group will be performed separately with the same methodology used for the primary endpoint.

Comparison of the change between the baseline and the 6-month visit for the primary endpoint and secondary endpoints between each experimental group and the control group will be performed separately using a linear mixed model. In this model, we will include the 2 measurements (at 2 and 6 months) for the time effect, the group effect (experimental/control), an adjustment for baseline values and center, and a time × group interaction. A contrast with a 2.5% type I error will be used to compare the 6-month change between the experimental and the control group.

The efficacy of the 2 therapeutic programs (face-to-face versus digital support) at 2 and 6 months will be compared using an analysis of covariance at the 5% significance level to compare the variations in the STAI score between the groups, adjusting for the baseline value.

VAS satisfaction and consumption of tobacco, alcohol, and drug use will be analyzed in each group using descriptive statistics.

The full version of the protocol can be viewed in Multimedia Appendix 4.

## Results

Recruitment started in January 2016. The duration of the inclusion period is 24 months, and the duration of research is 3 years (including 6 months for conducting the study with the last patients included and 6 months for data analysis). The final report will be written based on the CONSORT statement [48] and its adaptation for an eHealth trial [68] (Multimedia Appendix 5).

As of this writing, there have been no major changes during the study (eg, staff turnover, equipment breakdowns). We have lost a subject during the protocol. We are currently making an attrition or engagement diagram of the subjects over time that we will include in the final article once we have the results of the study.

To our knowledge, this is the first French study to examine the effectiveness of a computer-based stress management program (CBT) for patients with ADA.

## Discussion

The aim of this study is to demonstrate the efficacy of 2 therapeutic programs with 120 patients suffering from ADA and to compare these programs. To date, we have 50 of the required 120 participants (42%).

We can already cite as a limitation that because this is an eHealth study, the participants are not blinded to the treatment group.

Some elements of this randomized controlled trial would be different if it were conducted in routine clinical practice. Indeed, currently, patients come to do their eLearning sessions in consultation, so one can easily control adherence, which we would not be able to do if it were in clinical practice. This adherence is reinforced by minimal contact time with a nurse before each session (which is very short).

In clinical practice, a way to assess and reinforce adherence should be found. This issue is why we plan to improve this program by integrating tools from new information and communication technologies (eCBT, mCBT, eCoaching, telemedicine) and rely on a recent literature review concerning adherence to self-help treatment with digital media [37].

We would propose that the program is not just for the psychiatric population (tertiary prevention) but for a wider population exposed to stress who may suffer from stress-related disorders (primary and secondary prevention).

This program would make it easier to access treatment with the aim of preventing stress, which is not available in France at the present time.

## Appendix

Multimedia Appendix 1 Declaration to Commission Nationale de l’Informatique et des Libertés.

Multimedia Appendix 2 Patient information and informed consent.

Multimedia Appendix 3 Screenshots of the Seren@ctif program.

Multimedia Appendix 4 Full version of the protocol.

Multimedia Appendix 5 CONSORT-HEALTH check list.

## Abbreviations

ADA: adjustment disorder with anxiety
BDI: Beck Depression Inventory
CBT: cognitive behavioral therapy
eCRF: electronic case report form
DSM-5: Diagnostic and Statistical Manual of Mental Disorders, Fifth Edition
HADS: Hospital Anxiety and Depression Scale
iCBT: CBT program using digital support
ITT: intention-to-treat
MINI: Mini International Neuropsychiatric Interview
PSS: Perceived Stress Scale
PSWQ: Penn State Worry Questionnaire
SAP: statistical analysis plan
STAI-T: State-Trait Anxiety Inventory scale, trait subscale
VAS-stress: visual analog scale–stress
WLC: wait list control
