# Supplementary material for: Efficacy of Seren@ctif, a Computer-Based Stress Management Program for Patients With Adjustment Disorder With Anxiety: Protocol for a Controlled Trial
Source: JMIR Res Protoc. 2017 Oct 2;6(10):e190. doi: 10.2196/resprot.7976 (PMC5643843; doi:10.2196/resprot.7976)
Supplement: Multimedia Appendix 1 [file resprot_v6i10e190_app1.pdf]

8 rue de Vivienne - 75083 PARIS cedex 02
T. 01 53 73 22 22 - F. 01 53 73 22 00
www.cnil.fr

RÉCÉPISSÉ DE DÉCLARATION DE CONFORMITÉ À UNE MÉTHODOLOGIE DE RÉFÉRENCE

Numéro de déclaration

1531100 v 0

du 08-09-2011

Monsieur DERAEDT Guillaume
CENTRE HOSPITALIER REGIONAL
UNIVERSITAIRE DE LILLE
DIRECTION GENERAL
2 AVENUE OSCAR LAMBRET
59000 LILLE

Organisme déclarant

Nom : CENTRE HOSPITALIER REGIONAL UNIVERSITAIRE DE LILLE

Service :

Adresse : 2 AVENUE OSCAR LAMBRET

Code postal : 59000
Ville : LILLE

N° SIREN ou SIRET :
265906719 00017

Code NAF ou APE :

Tél. : 0320444366
Fax. :

Traitement déclaré

Finalité : MR-001 - Recherches biomédicales

Transferts d'informations hors de l'Union européenne : Non

La délivrance de ce récépissé atteste que vous avez effectué une déclaration de votre traitement à la CNIL et que votre dossier est formellement complet. Vous pouvez mettre en œuvre votre traitement. Cependant, la CNIL peut à tout moment vérifier, par courier ou par la voie d'un contrôle sur place, que ce traitement respecte l'ensemble des dispositions de la loi du 6 janvier 1978 modifiée en 2004. En tout état de cause, vous êtes tenu de respecter les obligations prévues par la loi et notament :

1) La définition et le respect de la finalité du traitement,
2) La pertinence des données traitées,
3) La conservation pendant une durée limitée des données,
4) La sécurité et la confidentialité des données,
5) Le respect des droits des intéressés : information sur leur droit d'accès, de rectification et d'opposition.

Pour plus de détails sur les obligations prévues par la loi « informatique et libertés », consultez le site internet de la CNIL : « www.cnil.fr »

Fait à Paris, le 12 septembre 2011
Par délégation de la commission

Alex Türk

Alex TÜRK
Président
