# Supplementary material for: Efficacy of Seren@ctif, a Computer-Based Stress Management Program for Patients With Adjustment Disorder With Anxiety: Protocol for a Controlled Trial
Source: JMIR Res Protoc. 2017 Oct 2;6(10):e190. doi: 10.2196/resprot.7976 (PMC5643843; doi:10.2196/resprot.7976)
Supplement: Multimedia Appendix 2 [file resprot_v6i10e190_app2.pdf]

Note d'information pour les participants à la recherche biomédicale intitulée
« Efficacité de deux programmes thérapeutiques de gestion du stress self-help dans le trouble de l'adaptation avec anxiété (TAA) : sur support numérique ou face à face avec un thérapeute versus groupe contrôle »

Promoteur : CHRU de Lille

Investigateur coordonnateur : Dr Dominique SERVANT
Service de Psychiatrie Adulte Hôpital Fontan
Rue André Verhaeghe 59 037 Lille cedex, France Tél: 03.20.44.44.60

Madame, Monsieur,
Le présent document décrit l'étude à laquelle il vous est proposé de participer. Il résume les informations actuellement disponibles en répondant aux différentes questions que vous pouvez vous poser dans le cadre de votre participation à cette recherche.

1) Pourquoi me propose t-on de participer à cette étude?

Nous souhaitons vous proposer de tester l'efficacité de deux programmes thérapeutiques de gestion du stress appelés « self-help » délivré soit par un thérapeute soit par support numérique avec un temps de contact limité. Le principe de ces programmes repose sur la gestion de l'anxiété par soi-même et ont pour objectif de vous apprendre les moyens de pouvoir changer par vous-même.

2) Quel est l'objectif de la recherche ?

L'objectif de cette étude est de montrer que chacun de ces deux programmes est efficace dans le traitement de l'anxiété, et de les comparer également entre eux.

3) Comment va se dérouler la recherche ?

Si vous acceptez de participer à cette étude, vous aurez une première consultation avec un investigateur d'environ 1 heure 15 qui consistera essentiellement en un entretien et plusieurs auto-questionnaires visant à évaluer votre niveau d'anxiété, d'humeur (c'est-à-dire votre moral) et de stress.
Si vous êtes enceinte vous ne pouvez participer à l'étude et si vous avez un doute un recueil d'urine pour réaliser un test de grossesse sera réalisé.
Ensuite, le médecin investigateur vous attribuera le traitement qui sera choisi par tirage au sort à partir d'un numéro pré établi pour que cette attribution se fasse au hasard, indépendamment des choix de votre médecin et de vous-même.

- Soit vous bénéficierez d'un programme self-help en présentiel avec un thérapeute qui vous sera proposé dans les semaines qui suivent cette première consultation en 5 séances individuelles de gestion du stress avec un thérapeute à raison d'une séance par semaine dans le centre où vous avez été inclus. Chaque séance dure environ entre 30 et 45 minutes. A la fin de chaque séance, vous aurez des exercices à réaliser chez vous afin d'acquérir les outils nécessaires pour gérer son stress. Vous devrez noter sur un carnet les dates et heures pendant lesquels vous avez fait ces exercices. Vous aurez 1 chance sur 3 de bénéficier de ce programme.

- Soit vous bénéficierez d'un programme self-help sur support informatique qui consistera dans les semaines qui suivent en 5 séances de gestion du stress devant un ordinateur dans le centre où vous avez été inclus et à raison d'une séance par semaine. Chaque séance dure entre 1 heure et 1 heure 15 minutes. Avant chaque séance vous aurez un temps de contact avec un membre de l'équipe soignante afin de répondre aux éventuelles questions, faire un bilan des exercices réalisés au domicile, vous expliquer le déroulement de la séance et éventuellement vous guider dans la navigation du programme informatique. A la fin de chaque séance, vous aurez un nouveau temps contact avec un membre de l'équipe soignante pour savoir si tout s'est bien déroulé et vous aurez une clé USB pour pouvoir faire les exercices de gestion du stress chez vous. Vous devrez noter sur un carnet les dates et heures pendant lesquels vous avez fait ces exercices. Vous aurez 1 chance sur 3 de bénéficier de ce programme.

- Soit vous bénéficierez tout d'abord de la prise en charge classique par votre médecin traitant et dans un deuxième temps de la thérapie de gestion du stress en self help. Un rendez-vous de consultation avec un thérapeute spécialisé vous sera proposé dans 2 à 4 mois (variable selon les délais de chaque centre). Vous pourrez alors choisir l'un des deux modes de thérapie que vous souhaitez avec le thérapeute ou sur support numérique. Avant de débuter la thérapie, vous aurez une visite à 2 mois avec un membre de l'équipe de recherche d'environ 50 minutes pour réévaluer vos niveaux d'anxiété et de stress et vos symptômes anxio-dépressifs par les mêmes autoquestionnaires que lors de la première visite. Vous avez 1 chance sur 3 d'avoir ce programme.

Pour tous les participants, vous serez revu à 2 mois et 6 mois après l'inclusion par un membre de l'équipe de recherche environ 50 minutes pour réévaluer le niveau d'anxiété et de stress et des symptômes anxio-dépressifs par les mêmes autoquestionnaires que lors de la première visite.

Tous les renseignements recueillis au cours des entretiens resteront **STRICTEMENT CONFIDENTIELS**, et seules les personnes collaborant au projet seront autorisées à les consulter. Ces données pourront être auditées ou inspectées par des personnes soumises au **secret professionnel**. Si vous acceptez de participer à cette étude, les résultats de cette étude seront **ANONYMES**.

La durée totale de votre participation à cette recherche sera de 6 mois.

4) Que se passera-t-il à la fin de ma participation à cette recherche ?

A la fin de votre participation, vous bénéficierez du suivi approprié à votre situation si nécessaire.

5) Quels sont les bénéfices attendus ?

Le bénéfice de cette étude est thérapeutique et elle devrait vous permettre d'acquérir des outils de gestion du stress et de l'anxiété, et ainsi pouvoir réduire votre niveau d'anxiété face à des situations stressantes. Il s'agit de pouvoir offrir un programme de soins complémentaire. Les patients inclus dans le groupe du programme de prise en charge classique par leur médecin généraliste bénéficieront également du programme de gestion du stress proposé dans l'étude. Ils auront le libre choix entre le mode avec thérapeute ou sur support numérique. Les centres leur proposeront ce programme psycho éducatif dans un délai de 2 à 4 mois. Ce délai est celui habituellement retrouvé dans les rares centres spécialisés français. Il n'y aura donc pour aucun patient participant à l'étude aucune perte de chance dans la prise en charge.

6) La recherche comporte-t-elle des risques, des effets indésirables et/ou des contraintes particulières ?

Les risques envisageables sont un éventuel impact psychologique négatif des questionnaires psychologiques réalisés.

7) Existe-t-il des alternatives au traitement/à la prise en charge ?

Le traitement habituel proposé et recommandé dans l'anxiété est la gestion du stress par des thérapies similaires et/ou par prescription de psychotropes. Si vous ne participez pas à cette étude, vous bénéficierez alors de la prise en charge habituellement pratiquée dans le centre, en tenant compte des délais habituels du centre.

8) Quelles sont les conditions de participation à la recherche ?

Afin de pouvoir participer à cette étude, vous devez être affilié(e) à un régime obligatoire d'Assurance Maladie ou ayant droit d'un assuré social.
Votre participation à cette étude nécessite que nous informions votre médecin traitant, sauf si vous le refusez.
Vous devez posséder un ordinateur (PC ou Mac) et pouvoir visionner des fichiers informatiques sous format classique (vidéo, PDF, power point).

Si vous acceptez de participer à cette étude, vous ne pourrez pas participer à une autre étude pendant les 2 mois qui suivent votre inclusion dans l'étude.

9) Quels sont mes droits en tant que participant à la recherche ?

Vous êtes libre d'accepter ou de refuser de participer à cette recherche sans avoir à vous justifier et sans que cela ne modifie la relation de soin existant avec l'équipe médicale vous prenant en charge.
Vous n'êtes pas obligé de nous donner votre décision tout de suite ; vous disposez du temps que vous estimez nécessaire pour prendre votre décision.

En cas d'acceptation, vous pourrez à tout moment revenir sur votre décision, sans nous en préciser la raison, et sans que cela n'altère la qualité des soins qui vous sont dispensés.
Par ailleurs, vous pourrez obtenir au cours ou à l'issue de la recherche, communication de vos données de santé détenues par votre médecin.
Dans le cadre de la recherche, un traitement de vos données personnelles sera mis en œuvre pour permettre d'analyser les résultats de l'étude au regard de l'objectif de cette dernière qui vous a été présenté. A cette fin, les données médicales vous concernant ou tout autre type de données existantes seront transmises au Promoteur

de la recherche ou aux personnes ou société agissant pour son compte en France ou à l'étranger. Ces données seront identifiées par un numéro de code et vos initiales. Ces données pourront également, dans des conditions assurant leur confidentialité, être transmises aux autorités de santé françaises. Conformément aux dispositions de la loi relative à l'informatique, aux fichiers et aux libertés, vous disposez d'un droit d'accès et de rectification. Vous disposez également d'un droit d'opposition à la transmission des données couvertes par le secret professionnel susceptibles d'être utilisées dans le cadre de cette recherche et d'être traitées. Ces droits s'exercent auprès du médecin qui vous a proposé de participer à cette étude.

Si vous le souhaitez, vous obtiendrez communication des résultats globaux de l'étude à la fin de celle-ci qui vous sera proposée par courrier.

Vous n'aurez à supporter aucune charge financière supplémentaire du fait de votre participation à cette étude.

Pour chaque visite réalisée dans le cadre de l'étude, vous percevrez une indemnité forfaitaire de 45€ visant à vous dédommager des frais de déplacement.

10) Le CHRU de Lille est-il autorisé à réaliser ce type de recherche?

Oui, en application de la loi, cette étude a été autorisée par l'ANSM[1], le …./…./……….. ; elle a également reçu, le …./…./……….. , un avis favorable du Comité de Protection des Personnes Nord Ouest IV, organisme officiel et indépendant qui a vocation à protéger la sécurité des personnes qui se prêtent à la recherche.
En outre, le CHRU de Lille, en sa qualité de promoteur, a souscrit une assurance pour la réalisation de cette étude. (SHAM – contrat n°)

11) A qui dois-je m'adresser en cas de questions ou de problèmes ?

Vous pouvez poser toutes les questions que vous souhaitez, avant, pendant et après l'étude en vous adressant au coordonnateur de la recherche : Dr. Dominique Servant, Hôpital Fontan – CHRU Lille - Tél : 03 20 44 44 60

Vous pouvez également vous adresser à :

L'investigateur principal du centre :

Nom : …………………………………………………..….

Coordonnées : …………………………………………

En cas d'urgence, vous pouvez contacter : ……………………………………………………………………………

Nous vous remercions de parapher chaque page de ce document (réalisé en trois exemplaires), afin d'attester que vous l'avez lu et compris. Si vous êtes d'accord pour participer à cette étude, nous vous remercions également de bien vouloir donner votre consentement écrit en signant le formulaire ci-après.

Paraphe de l'investigateur

Paraphe de l'intéressé, et/ou du représentant légal (barrer mention inutile)

[1] ANSM: Agence Nationale de Sécurité du Médicament et des produits de santé. Autorité de santé ayant notamment pour mission d'autoriser, de suivre et de contrôler le déroulement de la recherche.

« Efficacité de deux programmes thérapeutiques de gestion du stress self-help dans le trouble de l'adaptation avec anxiété (TAA) : sur support numérique ou face à face avec un thérapeute versus groupe contrôle »

Promoteur : CHRU de Lille

Investigateur coordonnateur : Dr Dominique SERVANT
Service de Psychiatrie Adulte Hôpital Fontan
Rue André Verhaeghe 59 037 Lille cedex, France Tél: 03.20.44.44.60

Je soussigné(e) Mme, Mlle, M. (rayer les mentions inutiles)

Nom, Prénom : ....

accepte librement et volontairement de participer à la recherche biomédicale intitulée : ***« Efficacité de deux programmes thérapeutiques de gestion du stress self-help dans le trouble de l'adaptation avec anxiété (TAA) : sur support numérique ou face à face avec un thérapeute versus groupe contrôle »*** dont le CHRU de Lille est promoteur et qui m'a été proposée par le

Docteur/Professeur (nom, coordonnées)……………………………………………………………………...........

Etant entendu que :

- Le médecin qui m'a informé(e) et a répondu à toutes mes questions, m'a précisé que ma participation à cette étude est libre et que je peux arrêter d'y participer à tout moment en informant préalablement mon médecin,

- J'ai été clairement informé(e) des éléments suivants : But de la Recherche- Méthodologie- Durée de ma participation- Bénéfices attendus- Contraintes- Risques prévisibles,

- J'ai bien compris que pour pouvoir participer à cette recherche, je dois être affilié(e) à un régime de sécurité sociale ou être ayant-droit d'un assuré social. Je confirme que c'est bien le cas,

- Si je le souhaite, je serai informé(e) par le médecin des résultats globaux de cette recherche selon les modalités figurant dans la note d'information qui m'a été remise,

-Mon consentement ne décharge en rien le médecin et le promoteur de l'ensemble de leurs responsabilités et je conserve tous mes droits garantis par la loi.

-J'accepte que les données enregistrées à l'occasion de cette recherche puissent faire l'objet d'un traitement informatisé par le promoteur ou pour son compte. J'ai bien noté que le droit d'accès prévu par la CNIL (loi du 6 janvier 1978 relative à l'informatique, aux fichiers et aux libertés (art. 39)) s'exerce à tout moment auprès du médecin qui me suit dans le cadre de la recherche et qui connaît mon identité. Je pourrai exercer mon droit de rectification et d'opposition auprès de ce même médecin, qui contactera le promoteur de la recherche.

Fait à …………………, le……………………	Fait à …………………, le……………………
Signature du participant :	Signature de l'investigateur ou de son représentant (barrer la mention inutile)

Le présent formulaire est réalisé en trois exemplaires, dont un est remis à l'intéressé, ou à son représentant légal (pour les majeurs sous tutelle ou sous curatelle).Un exemplaire sera conservé par l'investigateur ; le dernier sera conservé par le promoteur en toute confidentialité, conformément à la loi.
