# Supplementary material for: Efficacy of Seren@ctif, a Computer-Based Stress Management Program for Patients With Adjustment Disorder With Anxiety: Protocol for a Controlled Trial
Source: JMIR Res Protoc. 2017 Oct 2;6(10):e190. doi: 10.2196/resprot.7976 (PMC5643843; doi:10.2196/resprot.7976)

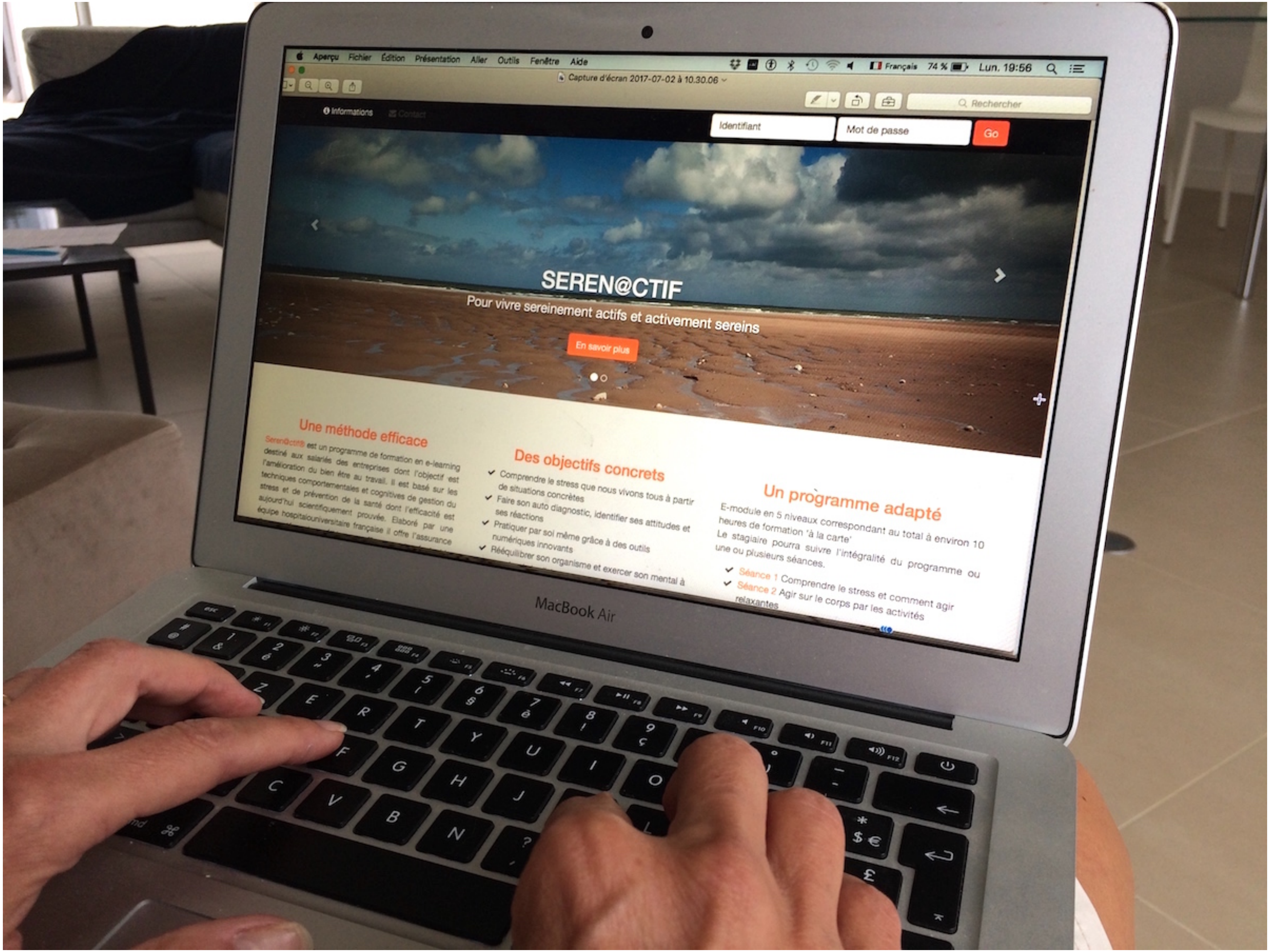
Aperçu Fichier Édition Présentation Aller Outils Fenêtre Aide
Français 74 % Lun. 19:56
Capture d'écran 2017-07-02 à 10.30.06
Rechercher
Informations
Identifiant
Mot de passe
Go
SEREN@CTIF
Pour vivre sereinement actifs et activement sereins
En savoir plus
Une méthode efficace
Des objectifs concrets
Comprendre le stress que nous vivons tous à partir de situations concrètes
Faire son auto diagnostic, identifier ses attitudes et ses réactions
Pratiquer par soi même grâce à des outils numériques innovants
Un programme adapté
E-module en 5 niveaux correspondant au total à environ 10 heures de formation 'à la carte'
Le stagiaire pourra suivre l'intégralité du programme ou une ou plusieurs séances.
Séance 1 Comprendre le stress et comment agir
Séance 2 Agir sur le corps par les activités relaxantes
MacBook Air

Presentation

Informations | Contact | Identifiant | Mot de passe | Go

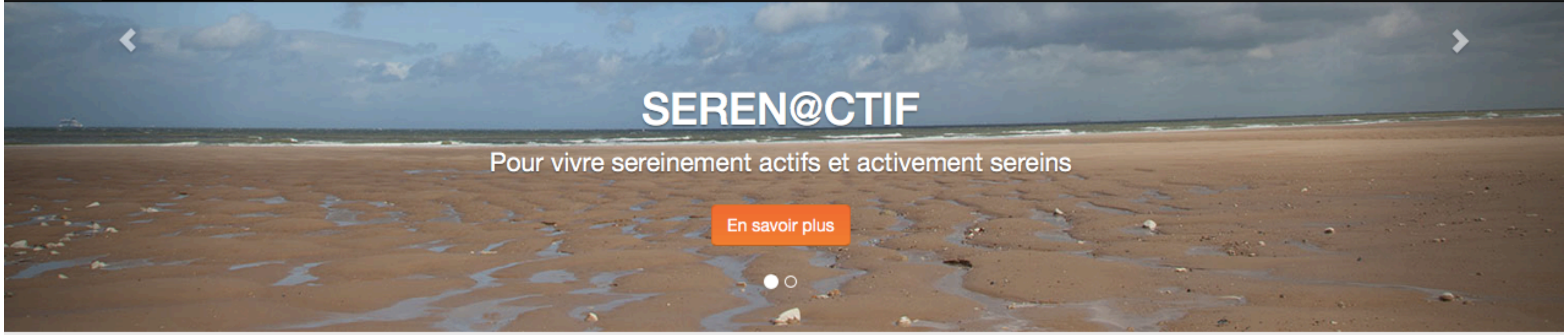

Une méthode efficace

Seren@ctif® est un programme de formation en e-learning destiné aux salariés des entreprises dont l'objectif est l'amélioration du bien être au travail. Il est basé sur les techniques comportementales et cognitives de gestion du stress et de prévention de la santé dont l'efficacité est aujourd'hui scientifiquement prouvée. Elaboré par une équipe hospitalouniversitaire française il offre l'assurance d'une démarche éthique, efficace et évaluable d'interventions pour améliorer la vie professionnelle et personnelle. Ce module proposé en elearning apporte flexibilité et gain de temps, ce qui permet de l'aborder selon son rythme et de mettre efficacement en application les acquis.

Des objectifs concrets

- Comprendre le stress que nous vivons tous à partir de situations concrètes
- Faire son auto diagnostic, identifier ses attitudes et ses réactions
- Pratiquer par soi même grâce à des outils numériques innovants
- Rééquilibrer son organisme et exercer son mental à prendre de la distance
- Mieux agir face aux problèmes et surmonter les difficultés.
- Développer des attitudes et des émotions positives pour soi et les autres

Un programme adapté

E-module en 5 niveaux correspondant au total à environ 10 heures de formation 'à la carte'
Le stagiaire pourra suivre l'intégralité du programme ou une ou plusieurs séances.

- Séance 1 Comprendre le stress et comment agir
- Séance 2 Agir sur le corps par les activités relaxantes
- Séance 3 Entrainer son mental pour moins ruminer
- Séance 4 Prendre conscience du moment présent
- Séance 5 S'exposer et adopter des attitudes positives

Summary

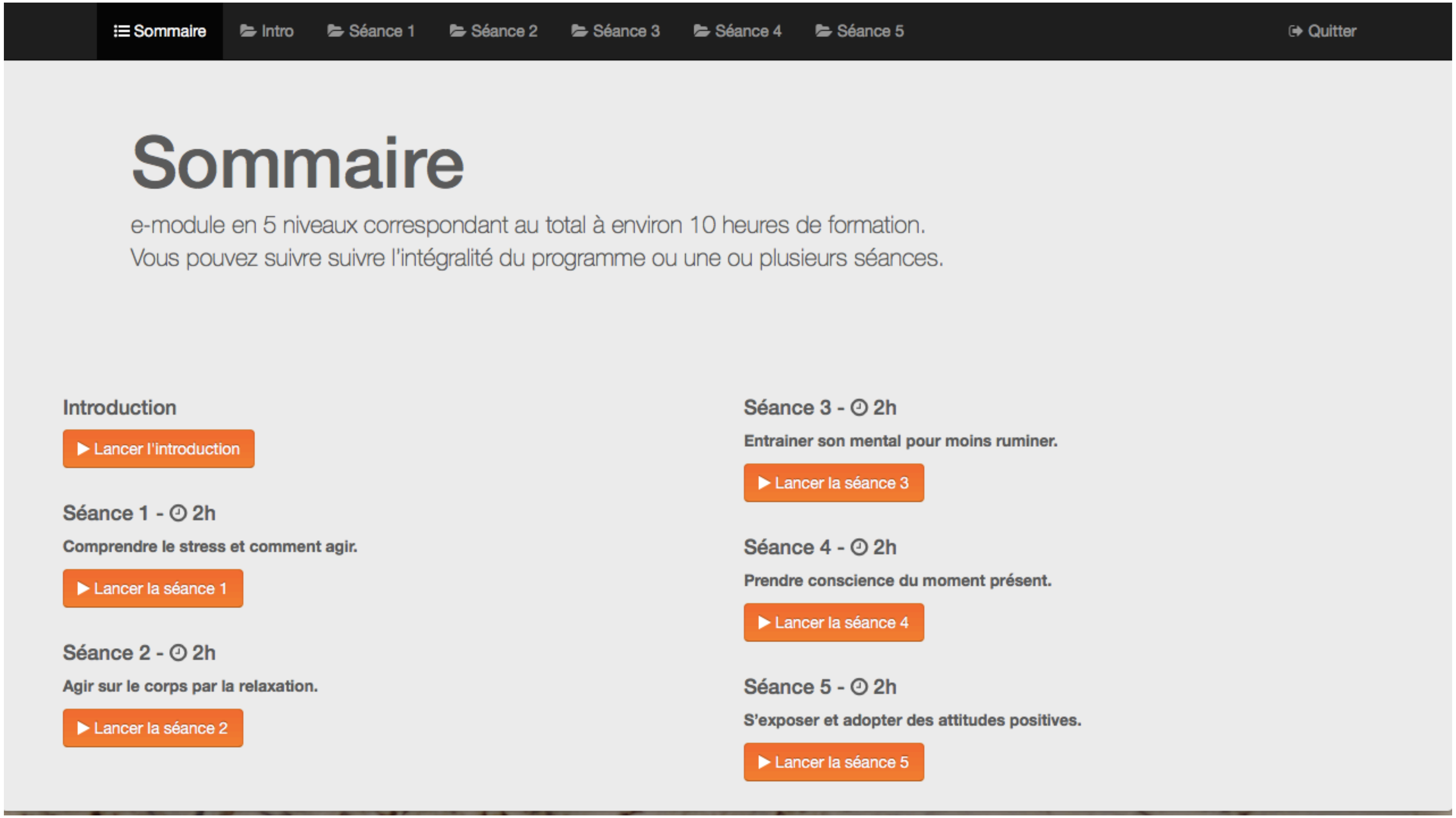
Sommaire
Intro
Séance 1
Séance 2
Séance 3
Séance 4
Séance 5
Quitter
Sommaire
e-module en 5 niveaux correspondant au total à environ 10 heures de formation.
Vous pouvez suivre suivre l'intégralité du programme ou une ou plusieurs séances.
Introduction
Lancer l'introduction
Séance 1 - 2h
Comprendre le stress et comment agir.
Lancer la séance 1
Séance 2 - 2h
Agir sur le corps par la relaxation.
Lancer la séance 2
Séance 3 - 2h
Entrainer son mental pour moins ruminer.
Lancer la séance 3
Séance 4 - 2h
Prendre conscience du moment présent.
Lancer la séance 4
Séance 5 - 2h
S'exposer et adopter des attitudes positives.
Lancer la séance 5

Videos

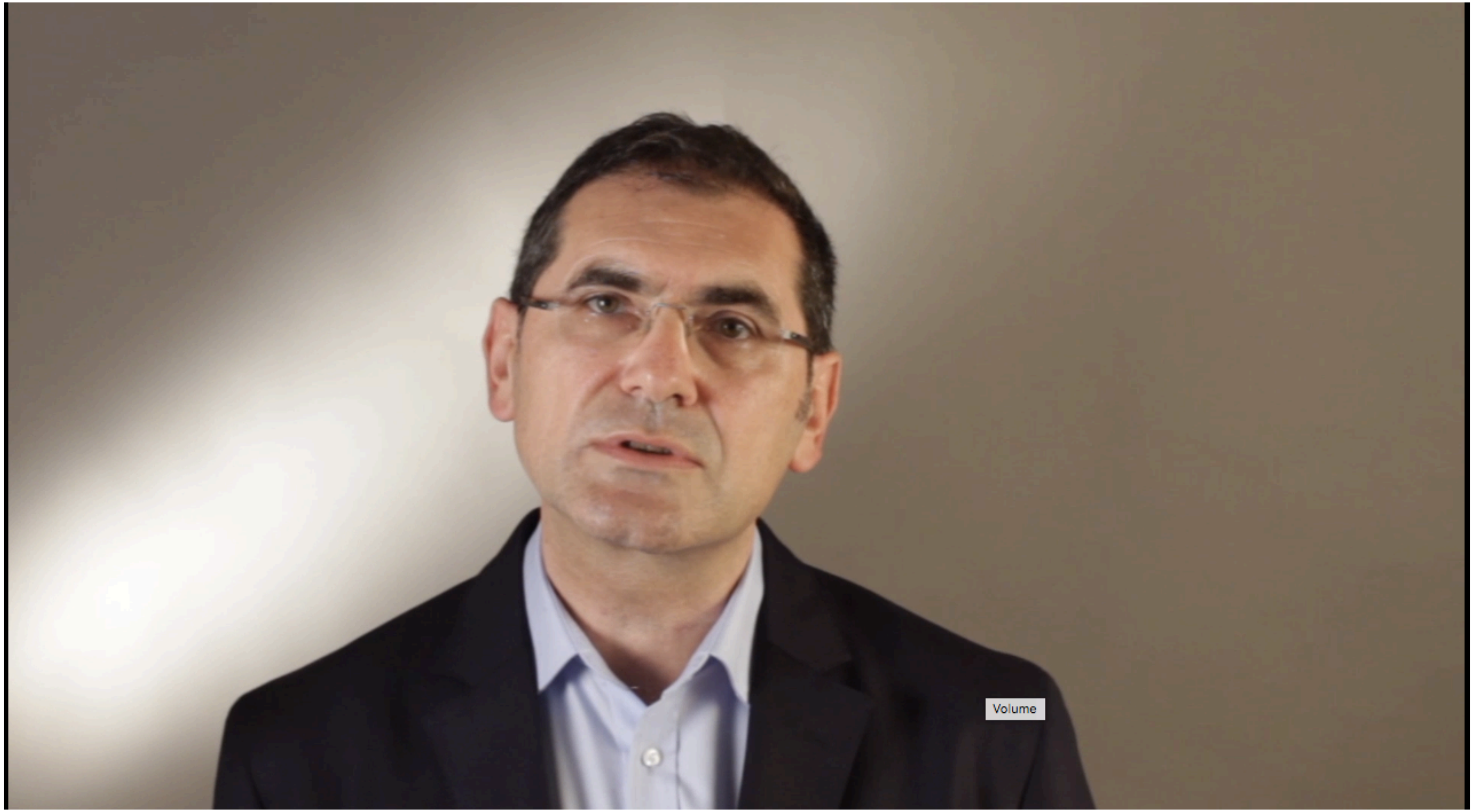

Session 2

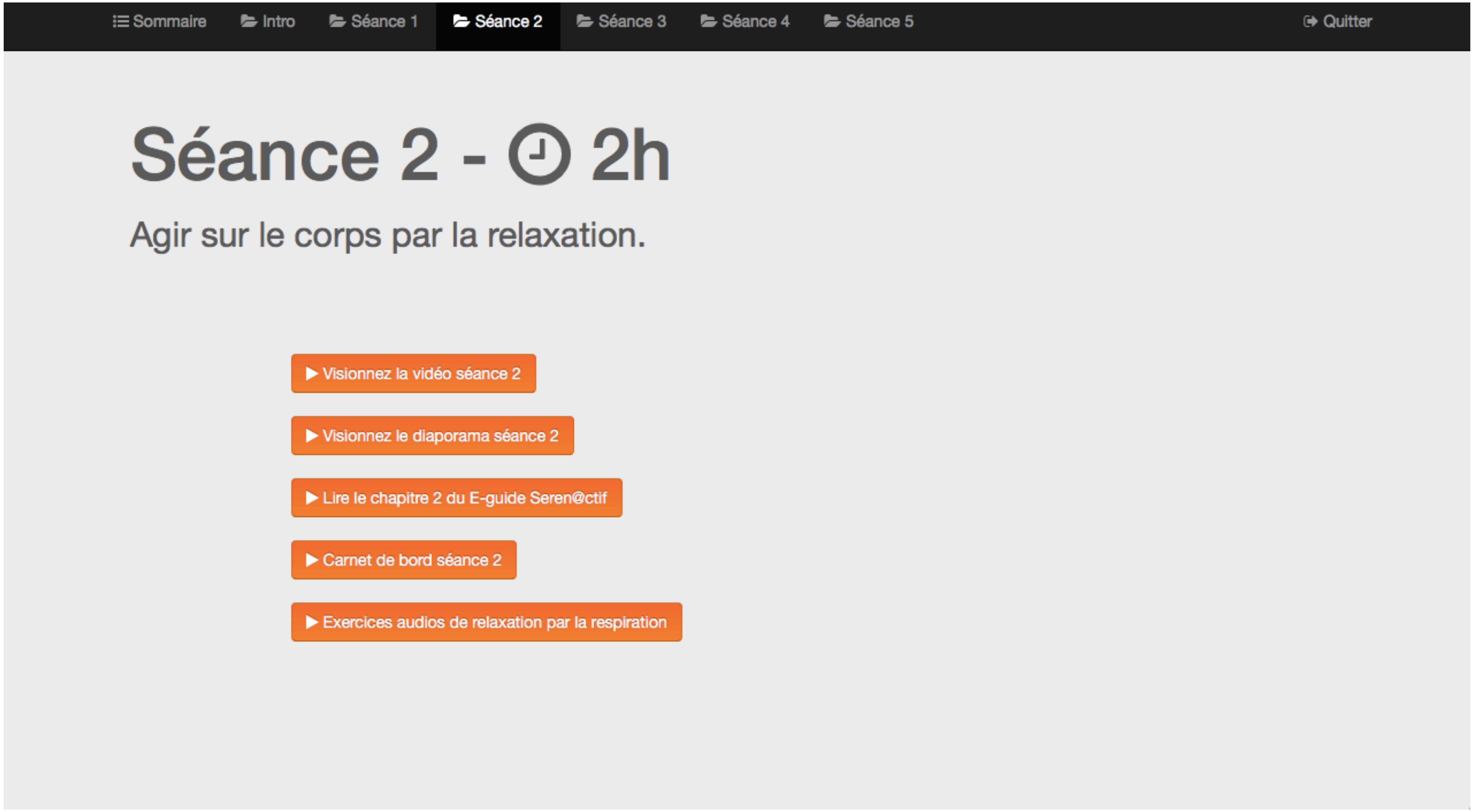
Sommaire
Intro
Séance 1
Séance 2
Séance 3
Séance 4
Séance 5
Quitter
Séance 2 - 2h
Agir sur le corps par la relaxation.
Visionnez la vidéo séance 2
Visionnez le diaporama séance 2
Lire le chapitre 2 du E-guide Seren@ctif
Carnet de bord séance 2
Exercices audios de relaxation par la respiration

Auto evaluation

Sommaire | Intro | Séance 1 | Séance 2 | Séance 3 | Séance 4 | Séance 5 | Quitter

Testez-vous avec le questionnaire Depistress

Ce test de dépistage simple est issu du programme Depistress®. L'objectif est de vous aider à auto évaluer votre niveau de stress, d'en repérer les principales sources et au terme de ce bilan de vous apporter quelques conseils pour mieux y faire face.

Depuis les 7 derniers jours, vous sentez-vous ?

Pas du tout stressé(e) — Extrêmement stressé(e)

Parmi ces différents domaines de votre vie quels sont ceux qui contribuent à votre niveau de stress ?

Budget et l'argent

Pas du tout stressé(e) — Extrêmement stressé(e)

Conditions de logement et d'habitat

Pas du tout stressé(e) — Extrêmement stressé(e)

Couple et vie sentimentale

Pas du tout stressé(e) — Extrêmement stressé(e)

Environnement

Pas du tout stressé(e) — Extrêmement stressé(e)

Famille

E-book Session 3

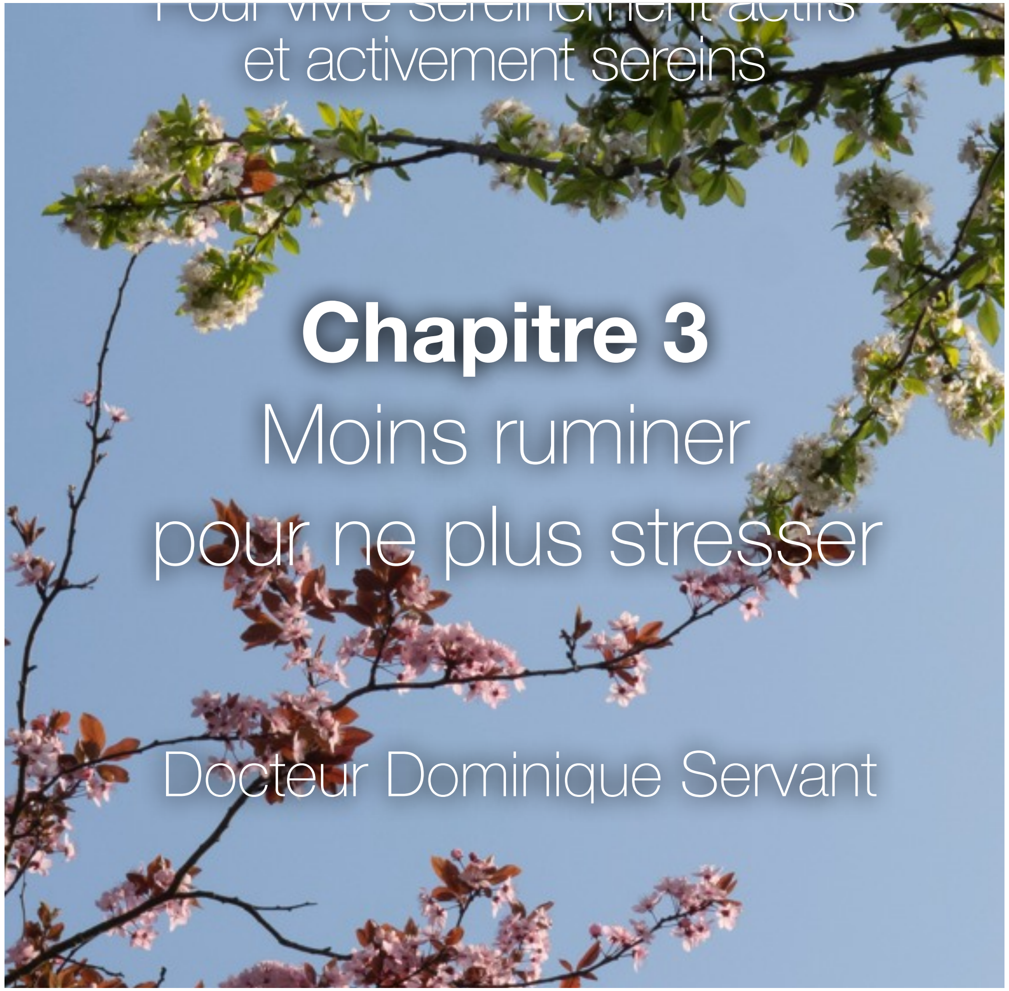

Log book session 3

Carnet de bord

Seren@ctif

Pour vivre sereinement actifs et activement sereins

Séance 3

Exercer son mental
pour moins stresser

Audiotapes Mindfulness exercices

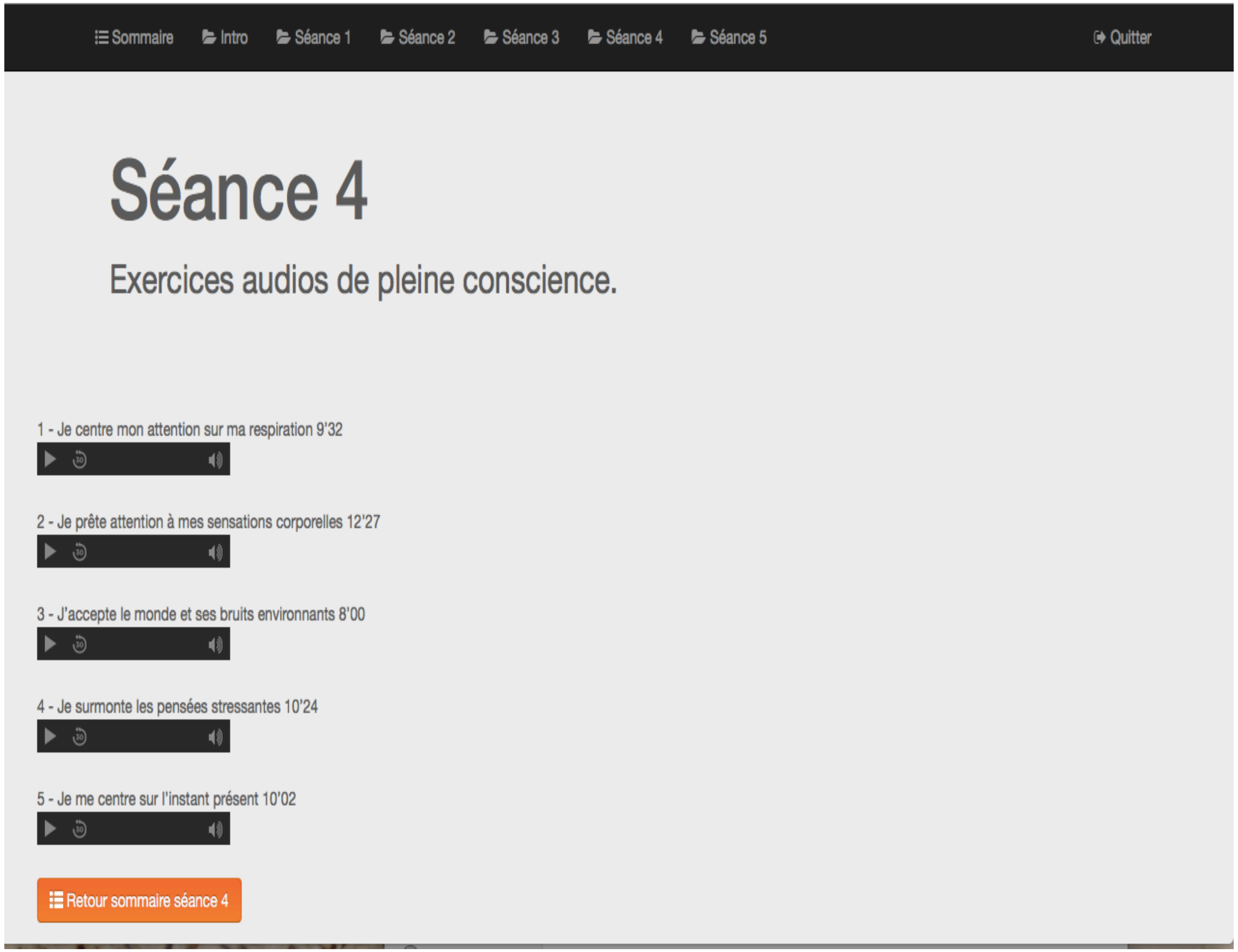

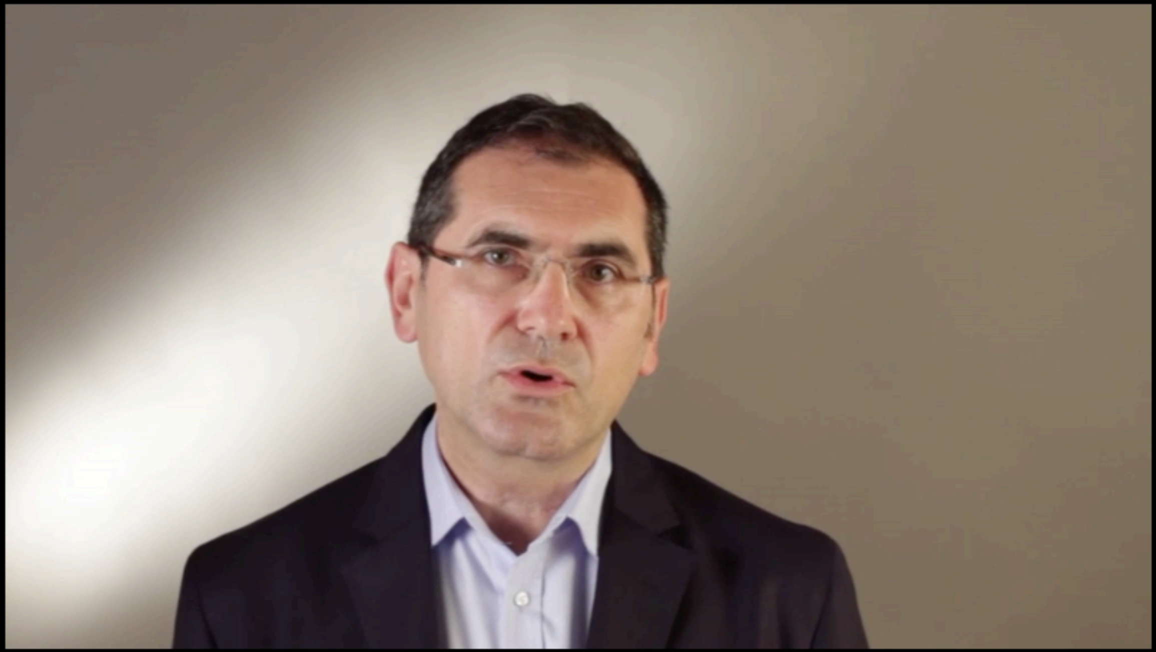

Supplement: Multimedia Appendix 3 [file resprot_v6i10e190_app3.pdf]
