# Supplementary material for: Efficacy of Seren@ctif, a Computer-Based Stress Management Program for Patients With Adjustment Disorder With Anxiety: Protocol for a Controlled Trial
Source: JMIR Res Protoc. 2017 Oct 2;6(10):e190. doi: 10.2196/resprot.7976 (PMC5643843; doi:10.2196/resprot.7976)
Supplement: Multimedia Appendix 4 [file resprot_v6i10e190_app4.pdf]

Centre Hospitalier Régional
Universitaire de Lille

Efficacy of two therapeutic self help stress management program for patients with Adjustment Disorder with Anxiety (ADA): conducted via e-learning or by face-to-face interview versus waiting list control group

Short Title: Seren@ctif study

Principal Investigator :
Dominique Servant (MD, PhD),
Service Universitaire de Psychiatrie
Hôpital Fontan rue André Verhaeghe
59037 Lille Cedex France
Phone : +33(0)320444460 Email : dominique.servant@chru-lille.fr

Sponsor of this study :
CHRU de Lille (University Hospital of Lille)
Délégation à la Recherche Clinique et à l’Innovation (DRCI)
6, rue du Pr Laguesse
59037 Lille Cedex France
Phone : +33(0)3 20 44 59 69

Investigators :

Dominique Servant MD, PhD (Principal investigator)
Service Universitaire de Psychiatrie du Pr Guillaume Vaiva
Hôpital Fontan rue André Verhaeghe
59037 Lille Cedex France
Phone : +33(0)320444460 Email : dominique.servant@chru-lille.fr

Pascal Delamillieure MD, PhD
Centre Esquirol, CHU DE Caen – Avenue Côte de Nacre –
14033 CAEN Cedex France
Phone : +33(0)231063106 Email : delamillieure-p@chu-caen.fr

Cyrille Guillaumont MD, PhD
Centre Hospitalier Philippe Pinel, route de Paris
80044 Amiens France
Phone : +33(0)322534646. Email : cguillaumont@ch-pinel.fr'

Methodology :

Alain Duhamel MD, PhD
Plateforme d'aide méthodologique (Centre d'Etudes et de Recherche en Informatique Médicale)
E.A. 2694
Université de Lille 2. Faculté de Médecine – Pôle recherche
59045 Lille Cedex France
Phone : +33(0)320446059 Email : alain.duhamel@chru-lille.fr

TABLE OF CONTENTS

I Synopsis 4
II Rationale 8
III Objectives of the research 10
IV Design/Methodology 10
4.1 Experimental design 10
4.2 Outcomes 11
4.3 Population 13
4.3.1 Inclusion Criteria 13
4.3.2 Non Inclusion Criteria 13
4.4 Evaluation Criteria 14
4.5 Sample Size 14
4.6 Statistical Analysis ..... **Erreur ! Signet non défini.**
V Logistics of the study 16
5.1 Research Teams involved in the study 16
5.2 Site participating in the study 17
5.3 Conduct of the study 17
5.4 Lenght of the study 21
5.5 Study stopping rules 22
5.6 Previous study - Exclusion period 22
5.7 Benefit/ Risk 22
5.8 Data Safety Monitoring Board 22
VI Stress Management Program 22
6.1 Stress management program face to face with a therapist Group A 23
6.2 Stress management program on digital support Group B 25
VII Adverse Events 26
VIII Access to data sources 27
IX Control and quality insurance 27
X Ethical and legal consideration 28
XI Data analyses and storage of research data documents 30
XII Study Funding and insurance 30
12.1 Funding 30
12.2 Insurance 30
XIII Publication- Valorisation 30
XIV Appendix 31
REFERENCES 31
Mini International Neuropsychiatric Interview MINI ..... **Erreur ! Signet non défini.**
Hospital Anxiety Depression Scale (HADS) 37
Penn-State Worry Questionnaire (PSWQ) 39
Spielberger Stait-Trait anxiety Inventory (STAI) 40
Visual Analogic Scale (VAS) 41
Perceived Stress Scale - PSS10 42
Beck Depression Inventory (BDI) 43

I Synopsis

SPONSOR OF THE STUDY	University Hospital of Lille
TITLE	Effectiveness of Two Stress Management Programs in patients with Adjustment Disorder With Anxiety (ADA) : Computer-based or Face- to -Face to Face Versus Control Group. Open Multicenter Prospective Randomized Controlled Therapeutic 3 Parallel Groups
PRINCIPAL INVESTIGATOR	Dominique SERVANT MD, PhD
NUMBER OF SITES	3 (Amiens, Caen, Lille) (France)
TYPE OF STUDY	Prospective multicentric controlled, open, randomized, with 3 parallel groups
OBJECTIVES	To demonstrate the efficacy of two cognitive-behavioral stress management programs, one conducted via e-learning and the other by face-to-face interviews, in patients responding to the diagnosis of Adjustment disorder with anxiety (ADA) in comparison to a control group of patients with ADA with only usual care by general practionner.<br><br>Secondary objectives:<br><br>- to compare the effectiveness at 2 months between both therapeutic programs.<br>- to evaluate the maintenance of the therapeutic effect at 6 months for both groups;<br>- to measure the clinical impact of both therapeutic programs on anxiety symptoms;<br>- to measure perceived stress and depressive symptoms at 2 months and 6 months;<br>- to measure the impact on the consumption of tobacco, alcohol, drug;<br>- to assess overall satisfaction.

EXPERIMENTAL DESIGN	3 parallel groups with balanced randomization :<br>- A group which benefits from a stress management program by face-to-face interviews. Group A : n=40 ;<br>- A group which benefits from a computer-based stress management program with minimal contact. Group B: n=40 ;<br>- A Waiting List Control group who receive usual healthcare by the general practionner. Group C : n=40.
CRITERIA	Main criterion<br>Change in the STAI scale between baseline and 2 month visit.<br><br>Secondary Criteria<br>- Change in the STAI scale between baseline and 6 month visit. Change in others validated self-report measure of anxiety and worry at 2 months and at 6 months (HADS anxiety subscale, PSWQ).<br>- Change in self validated self-report measures of stress at 2 months and at 6 months (VAS-stress, PSS)<br>- Change in self validated self-report measures of depression at 2 months and at 6 months (BDI, HADS depression subscale)<br>- Consumption of tobacco (number of cigarettes smoked per smoker per day), alcohol (number of drinks of alcohol per week, in France the standart drink is containing 10 grams of alcohol) and consumption and drug use (days per week for each product);<br>- VAS-satisfaction at 2 months and 6 months for the two therapeutic groups and at 6 month for the WLC group.
INCLUSION CRITERIA	- Ambulatory patient<br>- Male or female aged 18 to 60 years;<br>- Responding to the diagnosis of Adjustment Disorder with Anxiety (ADA) according to DSM-5 criteria;<br>- Patient currently not supported by structured psychotherapeutic treatment for ADA<br>- No new psychotropic drug therapy or stabilized for at least 3 months. In the latter case, the patient will be informed of the need to keep the same dosages for the duration of the study;<br>- Minimum score to the Hospital Anxiety and Depression scale (HADS anxiety subscale) greater than or equal to 10, maximum score at HADS depression subscale less than 10;<br>- Having access to a computer.

NON INCLUSION CRITERIA	- Subjects who does not read, or cannot use computer and computer support;<br>- Pregnant woman<br>- Persons incapable of consenting, or enjoying legal protection<br>- Persons deprived of liberty<br>- Patient with another psychiatric disorder according to the MINI (Mini International neuropsychiatric Interview) : Major Depressive Disorder (MDD), Obsessive Compulsive Disorder, Social Phobia, Panic Disorder with or without Agoraphobia, Post traumatic Disorder (PTSD), Alcool or substance Abuse or Disorder, Anorexia or Bulimia, Psychotic Disorder.<br>- Patients with suicide ideas (according to the MINI)
NUMBER OF PARTICIPANTS	120 patients are included :<br>40 patients in the group A<br>40 patients in the group B<br>40 patients in the group C
STATISTICAL ANALYSIS PLAN	The average change of the STAI score between the baseline (visit V0) and the visit at 2 months will be compared between each experimental arm and the standard arm separately. Each of the 2 tests will be performed at the 2.5% significance level (Bonferroni correction). We will use a covariance analysis adjusted on the STAI score at visit V0.
STUDY PROCEDURE	- Inclusion : Information and informed consent. Vérification des critères d'inclusion. Passation du MINI (diagnostic interview). Test de grossesse urinaire si femme en âge de procréer. Self reports STAI, HADS, QIPS, Perceived stress scale, BDI, VAS. Estimated duration : 1h15mn.<br><br>- Randomization in one of the 3 groups :<br><br>- Group A = patients following the stress management module guided by a therapist 5 sessions (1 per week) estimated duration : 45 minutes<br><br>- Groupe B = patients using a digital stress management 5 sessions (1 per week) estimated duration : 1h15 minutes for each session.<br><br>- Groupe C = a group of patients on waiting list and benefiting from the usual care through their general practionner. At the visit of 2 months patients choose one of the two self help treaments.<br><br>- Assesement at 2 months and 6 months :<br>Assesement of anxiety and depressive symptoms by self reports (STAI, HADS, PSWQ, PSS, BDI, VAS-stress).

	Adverse Evenements Monitoring Global Satisfaction (VAS).<br>The duration of each visit is estimated to be near 50 minutes.
ASSESSMENT OF THE BENEFITS AND THE RISKS ASSOCIATED WITH THE RESEARCH	Benefits:<br>The benefits of participating of this study for the patient suffering from ADA is : to learn self help stress managementand ; to reduce anxiety level ; to cope in a different way with stress in daily life.<br>Self help stress management program is recommended by the French National Authority for Health (HAS) to reduce benzodiazepine use in patients suffering from anxiety disorders.<br>All treatments are maintained (medical care and medication). This is a complementary measure beneficial to the patient.<br><br>Risks : none except an psychological impact during assesement by questionnaire.
CO-ENROLLEMENT AND EXCLUSION PERIOD	Co-enrollemet in a second study is prohibited before the assessment at 2 months.<br>Exclusion period is not necessary because the intervention is non invasive of and no medication is evaluated in this study.
INDEPENDENT MONITORING COMMITEE	Independant Safety Monitoring Commitee may not be needed :<br><br>- No drug medication<br>- No invasive intervention<br>- Insignificant impact of the study<br>- Expected Benefit for all the participants
DURATION OF THE STUDY	The duration of the study is 3 years.

II Rationale

Fifty years ago Hans Selye proposed the concept of stress as a non specific neuroendocrine response to a stressor agent (1). He developped the theory that stress is a major cause of medical problem particulary cardiovacular disease (2). Today stress means both stress factors and neurophysiologic and cognitive behavioral response (3).

Numerous studies have shown that exposure to a stressor increases the risk of psychiatric symptoms and disorders, especially anxiety (4). When anxiety symptoms are at a higher level than a normal reaction to a stressful event, we consider the possibility of a diagnosis of Adjustment Disorders, which are classified today under a stress-related category. They are responsible for significant direct and indirect costs from treatment, work stoppages and loss of productivity (5).
Ajustment Disorder with Anxiety (ADA) ADA is the most frequent and best characterized stress-related psychiatric disorder (6). According to the Diagnostic and Statistical Manual of Mental Disorders - fifth edition (DSM-5), a diagnosis of ADA is given following a psychosocial stressor or life event (e.g., divorce, job loss, serious physical illness), defined by anxiety occurring within 3 months following the occurrence of the event or stressful context (7) and with symptoms generally abating by 6 months after the event. Often considered a common disorder ranging from mild to moderate intensity, ADA is a true diagnostic entity. A general medical study showed that the level of anxiety is comparable to that of other anxiety disorders, such as Generalized Anxiety Disorder (GAD) (8).

Patients are generally not treated with a tailored approach but most often with medication, mainly benzodiazepines only (9). That rationale for such a prescription is a public health issue. Although stress management is considered the most appropriate psychological treatment for ADA, the evidence base for this approach is limited (10). In contrast, several studies compared Cognitive Behavioral Therapy (CBT) with other treatments for generalized anxiety disorders and found greater improvements in stress and anxiety symptoms from CBT. For example, in a study comparing CBT with relaxation, a post-treatment progression for State Trait Anxiety Inventory scale (STAI) scores from 53.04 to 46 and for Penn State Worry Questionnaire (PSWQ) scores from 61 to 51.13 were shown for the group that received CBT (11). Additionally, a study comparing psychodynamic psychotherapy with CBT found a greater improvement in the symptoms of stress and anxiety for the CBT intervention (a post-treatment progression of STAI scores from 58.83 to 43.41 and of PSWQ scores from 63.48 to 49.86 in this group) (12). Moreover, e-mental health options are considered uniquely suited for offering early intervention after a patient experiences stressful life events that can potentially trigger adjustment disorders.

It seems legitimate, given the prevalence and the human, economic and social costs of this pathology linked to stress, to develop such measures. It is not always possible to intervene upstream to reduce the exposure to stress and prevent the occurrence of ADA (primary prevention). Secondary and tertiary prevention measures are therefore useful to limit their consequences.

.

Stress management is a set of educational and psychotherapeutic measures that combines several cognitive-behavioral techniques. The aim is to allow the patient to reach a satisfactory level of emotional and cognitive control and to cope with stressors in order to reduce the negative consequences. The common practice is to

offer group sessions or individual interviews in the form of a structured module limited in time (3,13).
CBT has been shown to be an effective treatment for reducing anxiety symptoms in various somatic pathologies, such as cardiovascular disease (14,15) diabetes (16), chronic fatigue syndrome (17), and breast cancer (18) and in subjects with high levels of perceived stress or anxiety or burnout, particularly in the workplace (19). A meta-analysis (20) showed a moderate effect size (d = 0.53) of stress management programs in the context of work, which is considered an average effect size according to the Cohen criteria (19). For structured programs based on CBT, the effect is clearly greater (d = 1) (21).
Given that many patients do not have any access to stress and anxiety management programs, therapeutic education modules based on guides and self-help books have been offered and have shown positive results compared with classical CBT programs and control groups (22,23).

In recent years, the development of new technologies has enriched these self-help programs by integrating new tools (CD-ROM, USB key or directly accessible on the Internet), allowing better interactivity and use other than contact with the therapist for patients, which remains necessary for the program to be effective (24).
These computer-assisted programs are intended to limit the number and time of contact with a mental health professional (25,26,27). They were evaluated mainly in the general population, in student populations and in the corporate world in many countries, but unfortunately, they were not evaluated in France. The results showed an equivalent effectiveness to a stress management program via face-to-face interviews (28,29,30).
Recently, a meta-analysis evaluating the effect sizes of 26 computer- and Internet-based interventions was conducted on psychological stress, and found significant results in terms of reduction of symptoms of stress (Cohen d = 0.43 (95% CI 0.31-0.54), depression (Cohen d=0.34, 95% CI 0.21-0.48) and anxiety (Cohen d=0.32, 95% CI 0.17-0.47) compared to other types of interventions. These results provide evidence that web- and computer-based stress management interventions can be effective and have the potential to reduce stress-related mental health problems on a large scale (31).

It was in this context that Seren@ctif, a computer-based self-help management program, was developed at Lille University Hospital. It is the first French CBT program using digital support (iCBT).
A pilot study was carried out between January and June 2014 to study the feasibility of this therapeutic program (32). The results are satisfactory (average scores for satisfaction questionnaires are high, with scores ranging from 4 to 5 on a 5-point Likert scale). These results were the reason why it seemed appropriate to go further by evaluating this program in a controlled manner.

To our knowledge, this study is the first French study to examine the effectiveness of a computer-based stress management program for patients with Adjustment Disorder with Anxiety (ADA).
The Seren@ctif program may be useful within the framework of a psycho-educative approach. It could also be advised for people suffering from other diseases related to stress and for people with a clinical level of perceived stress.

III Objectives of the research

A) Main objective

The aim of this study is to assess the effectiveness of a 5-week standardized stress management program for reducing anxiety conducted via e-learning (iCBT) or through face-to-face interviews (CBT) with patients suffering from Adjustment Disorder with Anxiety (ADA) as defined by the Diagnostic and Statistical Manual of Mental Disorders - Fifth Edition (DSM-5) criteria for reducing anxiety compared with a waiting list control group (WLC). These patients seek treatment in a psychiatric unit for treating anxiety disorders at a university hospital. The primary outcome is the change in the State Trait Anxiety Inventory scale (STAI) between baseline and the two-month visit.

B) Secondary objectives

The secondary objectives of this study are (1) to evaluate the maintenance of effectiveness of the two therapeutic programs at 6 months; (2) to compare the change in stress, worry, anxiety and depressive symptoms in the two therapeutic stress management programs (iCBT and CBT) at 2 and 6 months; (3) to measure the impact of the two therapeutic programs on the consumption of tobacco, alcohol, and drug use; and (4) to assess overall satisfaction with the two therapeutic programs at 2 and 6 months and with the WLC at 6 months.
The secondary outcomes include the change in the STAI scores at 6 months, the Hospital Anxiety Depression Scale (HADS) anxiety subscale scores at 2 and 6 months, worry symptoms evaluated by the Penn State Worry Questionnaire (PSWQ), stress level evaluated by the Perceived Stress Scale (PSS) and the Visual Analogic Scale - Stress (VAS-stress) at 2 and 6 months, and depressive symptoms evaluated by the Beck Depression Inventory (BDI) and the HADS depression subscale at 2 and 6 months. Overall satisfaction is evaluated by the VAS-satisfaction at 2 and 6 months for the two therapeutic groups and at 6 months for the WLC group. The change in consumption of tobacco, alcohol and drugs is evaluated at 2 and 6 months.

IV Design/Methodology

4.1 Experimental design

It is a multicenter, comparative, prospective, unblinded, randomized, controlled study in 3 parallel groups. As it is not possible to mask the different treatment groups, patients were not blinded from their intervention group.

Patients were referred by their general practitioner to a psychiatric consultation service for psychological treatment of anxiety symptoms in a context of recent stress (occurred recently). To improve recruitment, general practitioners of the three areas were informed of the study by local investigator during continuing medical education (CME), scientific meetings and all types of collaborative contacts between primary care and psychiatry service. We also inform directly general practitioner who were involved in previous research on ADA and CBT and invit them to refer patients.

During a medical interview, the participants will have complete oral and written information detailing the progress of the trial. A newsletter will be given to the participant before it is included in the study, allowing the participant to have a period of reflection. Informed consent will be collected for each topic before they enter the study.

Each newly referred patient was asked to answer the optional adjustment disorder section (Adjustment disorder) of the Mini International Neuropsychiatric Interview (MINI) (33), French version [47], to check if he or she meets to the ADA criteria according the DSM-5. The MINI was administered by clinical investigators who are trained in psychiatry as a face-to-face interview in paper version.

Assessments are conducted for the 3 groups at baseline, 2 months and 6 months. At baseline, participants have to complete the self-administered questionnaires for anxiety and depressive symptoms, and care contacts and medications are recorded. At 2 and 6 months, an evaluation of overall satisfaction as well as adverse events is additionally carried out. Self-assessments were collected in the face-to-face sessions with the investigator.
It is the same psychiatrist investigator for each site who generated the random allocation sequence, enrolled the participants, assigned the participants to the interventions and assessed the participants, so he is not blinded to the intervention group.

Patients will be randomized in 3 groups :

- A group of patients following the stress management module guided by a therapist 5 sessions. 45 minutes weekly session. Group A

- A group of patients using a digital stress management module 5 sessions. 1h15 minutes weekly session. Group B

- A group of patients on waiting list and benefiting from the usual care through their general practionner Groupe C

At the visit of 2 months patients in the Group C choose one of the two self help treaments.

4.2 Outcomes

Interviews and self-assessments were collected face-to-face with the investigator.

Anxiety

- **Spielberger State Trait Anxiety Inventory (STAI)** - Form Y (Spielberger, Gorsuch, Lushene, Vagg & Jacobs, 1983. The French adaptation by Bruchon-Schweitzer & Paulhan, 1993) is a 20-item questionnaire with 4 levels of ratings from "not at all" rated 1 to "much" rated 4 (total score of 20 to 80), that captures how the subjects feel "generally" (9 reversed items) (26). Estimated duration to complete the questionnaire : 5 minutes (34).

- **The Hospital Anxiety and Depression Scale - HAD** (Sigmond and Snaith, 1983): This questionnaire queries anxiety and depressive disorders using 14

items rated on a 0 to 3 scale. Seven questions relate to anxiety (total A). Seven questions relate to depression (total D). A score between 8 and 10 on each of the sub-scales is considered a risk (possible or probable) of anxiety or depressive disorders. Estimated duration to complete the questionnaire : 3 minutes (35).

- **The Penn-State Worry Questionnaire (PSWQ)** (Meyer, Miller, Metzger, & Borkovec, 1990; traduction française par Gosselin, Dugas, Ladouceur, & Freeston, 2001). It is a self-assessment questionnaire, consisting of 16 items, measuring the general tendency to worry. The answers are based on a 5-point Likert scale ranging from "not at all characteristic" side (1) to "extremely characteristic" (5) (score range from 16 to 80) (36). Estimated duration to complete the questionnaire : 5 minutes.

Stress

- **The Perceived Stress Scale (PSS)** comes from the Stress Transactional Model. There are several versions with 14, 10 and 4 items and the one used in this study is the one with 14 items. The total score ranges from 0 to 56, and higher scores represent higher stress levels. Two dimensions emerge from this scale: perceived threat and perceived personal effectiveness (37). Estimated duration to complete the scale : 5 minutes.

- **The visual analogue scale of stress (VAS-stress)** is often used to measure the intensity of various symptoms, especially pain. It was used for the first time in 1996 for a subjective assessment of stress. The simplest VAS scale is a horizontal segment whose ends are defined as the limits of the parameter to be measured, oriented from the best towards the worst. A study found external validity for the French version of VAS-stress by comparing its scores with the PSS and it has a good sensitivity/specificity ratio (38). Estimated duration to complete the questionnaire : 1 minute.

Depression

The Hospital Anxiety and Depression Scale (HADS) (Sigmond and Snaith, 1983) assesses anxiety and depressive disorders using 14 items rated 0 to 3. Seven questions are relative to depressive symptoms (total D). A score between 8 and 10 on each of the sub-scales is considered a risk (possible or probable) of anxiety or depressive disorders (38).

The abbreviated Beck depression inventory (BDI) is a self-assessment questionnaire measuring the severity of the depression with 13 items rated from 0 to 39 (39). Estimated duration to complete the questionnaire : 5 minutes.

Interview for diagnosis of psychiatric disorders

The Mini International Neuropsychiatric Interview Version 5.0.0 (MINI; Sheehan et al., 1998) is a fully structured diagnostic interview that assesses a major axis for the diagnosis of disorders [46]. Each newly referred patient was asked to answer the optional adjustment disorder section of the MINI from the French version (33).

The MINI was administered by research assistants who were trained to established reliability criteria. Any participant who met diagnostic criteria for a DSM-5 Axis I diagnosis was excluded from the study. There was no major change between DSM-IV and DSM-5 for anxiety and depressive disorders.

Satisfaction

The visual analogue scale of satisfaction (VAS-satisfaction) measures the overall satisfaction with the program.

4.3 Population

4.3.1 Inclusion Criteria

- Being an ambulatory patient

- Being male or female aged 18 to 60 years; We use a cutoff age of 60 years for inclusion to limit chronic somatic comorbidities

- The patient must (3) receive a diagnosis of Adjustment Disorder with Anxiety (ADA) according to DSM-5 criteria (7);

- The patient must currently not be supported by structured psychotherapeutic treatment for ADA or for another problem,;

- The patient have no new psychotropic drug therapy or be stabilized for at least 3 months. In the latter case, the patient will be informed of the need to keep the same dosages for the duration of the study;

- The patient must have a minimum score on the Hospital Anxiety and Depression scale (HADS) anxiety subscale greater than or equal to 10 and a maximum score on the HADS depression subscale of less than 10;

- The patient have access to a computer.

4.3.2 Exclusion Criteria

- The subjects who cannot read (checked at the time of the self-assessment questionnaires)

- The subjects cannot use a computer or computer support (the platform is easy-to-use, and a nurse is available to guide the patient in the navigation of the computer program)

- The pregnant women, as recommended by the French ethics committee. If the subject is a woman, a urine pregnancy test is performed.

- The persons incapable of consenting, not having legal protection, or being deprived of liberty

- The patients with another psychiatric disorder according to the MINI (Mini International neuropsychiatric Interview): Major Depressive Disorder (MDD), Obsessive Compulsive Disorder, Social Phobia, Panic Disorder with or without Agoraphobia, Post traumatic Disorder (PTSD), Alcohol or substance Abuse or Disorder, Anorexia or Bulimia, Psychotic Disorder. Patients with suicide ideas (according to the MINI).

4.4 Evaluation Criteria

4.4.1 The main criteria

The main criteria is the Score at the Spielberger State Anxiety Inventory STAI at 2 months.

4.4.2 Secondary Criteria

- Spielberger State Anxiety Inventory STAI at 6 months.
- Hospital Depression and Anxiety Scale (HADS) score at 2 months and 6 months
- Beck Depression Inventory (BDI) Score at 2 months and at 6 months
- VAS-stress Score at 2 months and 6 months.
- Perceived Stress Scale score
- Penn-State Worry Questionnaire score at 2 and 6 mois
- Consumption and drug use, consumption of tobacco, alcohol and toxics
-
- VAS-satisfaction score at 2 months (Group A and B) at 6 months (Group A, B,C).

4.5 Sample Size

In a study on the clinical and psychometric characteristics of ADA, the mean (standard deviation) of the STAI baseline score was 52.1 (14.6) (8). Two clinical studies with patients with Generalized Anxiety Disorder one comparing CBT with relaxation (11) and the other comparing CBT with brief psychodynamic psychotherapy (12), showed changes in the STAI scores of 7 and 16, respectively. In view of these results, we hypothesize that an average difference between each experimental arm and the control arm of 11.5 (mean vof 7 and 16). To demonstrate this difference in variation, with a standard deviation of the STAI score change of 14.6 (assuming a conservative correlation coefficient of 0.5 between the baseline and 2-month STAI measures), a 2.5% type I error (using a Bonferroni correction for the 2 pre-specified comparisons) and a power of 80%, it is necessary to include 32 patients

per group. Although the primary analysis of the primary outcome will be adjusted for baseline values, the sample size calculation does not take into account this adjustment to maximize the power. Finally, considering 20% missing data, 120 patients (40 per group) are planned in this clinical trial.

Data collection and management

Data are recorded in an electronic case report form (eCRF) developed using Clinsight (ENNOV). The eCRF is used for data entry at each investigator site, and every center is responsible for the patient's anonymization. The eCRF was created, tested and validated before the start of data entry. The data necessary for monitoring the primary and secondary endpoints are identified and managed at regular intervals throughout the trial. Data are monitored by the data management team of the data management department of University Hospital of Lille using predefined data management rules, and queries are automatically edited. Finally, overall automated monitoring is performed by the data manager at the end of data entry. In case of discrepancies, queries are edited to resolve the problems encountered. After validation, the database is frozen and exported to the statistical software package for analysis.

Statistical analysis

Statistical analysis will be conducted in a blinded fashion with a blinded code for the intervention.
All statistical analyses will be carried out independently in the department of Biostatistics of the University Hospital of Lille. SAS 9.3 software or later will be used. P-values will be reported as the actual values, unless $P<0.001$. No interim analyses are planned. A detailed statistical analysis plan (SAP) will be drafted and validated before the database is frozen. Patient characteristics at baseline will be described for each of the three arms; the quantitative variables will be described either by the mean and the standard deviation in case of a Gaussian distribution or by the median and the interquartile if not. The normality of the distributions will be verified graphically by histograms and by the Shapiro-Wilk test. The qualitative variables will be described by the numbers and percentages of each category. All analyses for the primary and secondary objectives will be performed on all randomized patients in their original group of randomization according to intention to treat principles (ITT). No subgroup analysis will be performed.

Main objective

Comparison on the primary endpoint (2-month change in STAI scale) between each experimental group and the control group will be performed separately using an analysis of covariance (ANCOVA) with an adjustment for the STAI baseline value and the center. Since two comparisons will be made for the primary analysis, each of them will be performed at the 2.5% significance level (Bonferroni correction). The absolute mean differences and the effect sizes (standardized mean difference) will be reported with the 95% confidence interval. In case of deviation from normality of model residuals, nonparametric analysis will be used; absolute changes between baseline and 2-month visits will be calculated and compared between the experimental arm and control arm using non-parametric analysis of covariance adjusted for baseline values (40,41). The primary analysis will be conducted according to the ITT principle. Missing values will be handled by multiple imputation

procedures. Missing data will be imputed under the missing at random assumption using a regression switching approach (chained equation with m=20 imputations) with predictive the mean matching method for continuous variables and logistic regression (binary, ordinal or polynomial) for qualitative variables (42). The imputation procedure will be performed using the main baseline characteristics, the outcome and the variable “group of randomization”, and multiple imputed data sets will be combined using Rubin’s rules (43,44). Sensitivity analyses will be first conducted using all available STAI measurements (complete cases analysis) and, second, in patients without major deviation from protocol (per protocol analysis); major deviations will be specified in the SAP.

Secondary objectives

For the secondary endpoints of the HADS anxiety subscale, PSWQ, VAS-stress, PSS, BDI, and HADS depression subscale scores, comparisons of the changes between the baseline and the 2-month visit between each experimental group and the control group will be performed separately with the same methodology used for the primary endpoint.
Comparison of the change between the baseline and the 6-month visit for the primary endpoint and secondary endpoints between each experimental group and the control group will be performed separately using a linear mixed model. In this model, we will include the two measurements (at 2 and 6 months) for the time effect, the group effect (experimental / control), an adjustment for baseline values and center, and a time x group interaction. A contrast with a 2.5% type 1 error will be used to compare the 6-month change between the experimental and the control group.
The efficacy of the two therapeutic programs (in face-to-face versus digital support) at 2 and 6 months will be compared using an analysis of covariance at the 5% significance level to compare the variations in the STAI score between the groups, adjusting for the baseline value.
VAS satisfaction, consumption of tobacco, alcohol and drug use will be analyzed in each group using descriptive statistics.

These goals will be assessed by the following numerical scores: STAI score at 6 month, HADS score at 2 month and 6 month, VAS-stress score at 2 months and 6 months, PSS score at 2 months and 6 months, PSWQ score at 2 months and 6 months. We will use the same statistical methodology used for the main objective.

V Logistic of the study

5.1 Research Teans involved in the study

The study is coordonated by Dominique Servant MD, PhD, psychiatrist who holds the ‘Habilitation à Diriger les recherches’ (HDR). HDR is the higher Post Doctoral Degree from the french university who gives an Accreditation to Supervise Research. He has expertise in the field of psychopathology particularly stress and anxiety disorders. He is the head of coordinates the stress and anxiety unit in the Adult Psychiatry department of the University of Lille (France), headed by Professor Guillaume VAIVA. It is a consultation service offering an ambulatory care specifically dedicated to stress and anxiety.

5.2 Site participating in the study

Service de Psychiatrie Adulte
Hôpital Fontan
Consultation spécialisée sur le stress et l'anxiété
Rue André Verhaeghe 59037 Lille Cedex (France)
Phone : +33(0)320444460

Centre Hospitalier et Universitaire de Caen
Centre Esquirol
Service de Psychiatrie Adulte
Avenue Georges Clémenceau – 14033 Caen Cedex 9 (France)
Phone : +33(0)231063106

Centre Hospitalier Pinel
Service de Psychiatrie Adulte
Route de Paris – 80000 Amiens (France)
Phone : +33(0)322534646

5.3 Conduct of the study

The study is being conducted at three university hospitals (Lille, Amiens, Caen) in the northwest area of France as part of the university communities (InterRégional). Patients are referred by their general practitioner to a psychiatric consultation service for psychological treatment of anxiety symptoms in a context of recent stress (occurred recently). To improve recruitment, general practitioners in the three areas were informed of the study by a local investigator during continuing medical education (CME), scientific meetings and all types of collaborative contacts between primary care and psychiatry services. We also directly inform the general practitioners who were involved in previous research on ADA and CBT and invite them to refer patients.
During a medical interview, the participants will complete oral and written information detailing the progress of the trial. A newsletter will be given to the participant before it is included in the study, allowing the participant to have a period of reflection.
Informed consent will be collected for each subject before they enter the study.

5.3.1 Inclusion

The investigator will propose the study to any potentially inclusive patient referred for ADA in a psychiatric ambulatory service. During a medical interview, the subjects will have complete oral and written information detailing the progress of the trial. A newsletter will be given to the subject by the investigator before the inclusion in the study, allowing the participant to have a period of reflection. Informed consent will be collected for each participant before to be enrolled in the study. Protocol acts do not begin without the patient's signed agreement. The information letter and the consent form will be sign in triplicate, a copy of which will be given to the subject; A copy will be kept by the investigator who will transmit the last copy to the sponsor in a sealed envelope

Inclusion criteria and absence of exclusion criteria will be verified. A psychological interview will be conducted using the MINI. A urinary pregnancy test will be performed in women of childbearing age. The following self-questionnaires will be completed: STAI, HADS, PSWQ, PSS, VAS-stress, BDI. The socio-demographic characteristics of the subject will be recorded (age, sex, level of study, occupational activity), as well as the history and concomitant treatment. The lenght of this visit is estimated at about 1h15.

5.3.2 Randomisation and description of interventions for each group

Immediately after inclusion and assessment, patients are randomly allocated in a 1:1:1 ratio into 3 groups using a web-based central randomization: a group of patients using a digital stress management module (Group A), a group of patients following the stress management module guided by a therapist in attendance (Group B), and a group of patients on a waiting list and benefiting from usual care through their attending physician (Group C).
The randomization sequence is provided by an independent statistician (who does not take part in assessing the patients at any point in the study) using usingcomputer-generated random numbers with block sizes of six and center stratification consistent with the CONSORT standards (40,41). The randomization sequence is implemented in the electronic case-report form (eCRF) system to ensure a centralized, real-time randomization procedure.
A document describing the randomization procedure is kept confidential in the Clinical Investigation Centre of Lille University Hospital.

Stress management program

The content of the two programs is identical, but they differ in their materials (computer for one, face-to-face for the other).

It is based on standard CBT principles and includes five modules that include the following topics: information about stress and stress reaction and assessment, deep respiration and relaxation techniques, cognitive restructuration, mindfulness and acceptation, behavioral skills as solving problem, time management, healthy behaviors, and problem-solving and emotion regulation.

Stress management module guided by a face to face therapist: Group A

The program includes 5 weekly sessions lasting forty-five minutes or 1-hour with trained clinical psychologists (graduate of a Masters of cognitive and emotional therapy with a minimum of one year of practice in CBT and cognitive-behavioral stress management). Information, exercises, and homework assignments are delivered by the therapist without self-help support. At the beginning of each session, the therapist asks the patient about adverse events and changes in drug doses since the last session.

The Stress Management Program is based on a 10-session Stress and Anxiety Module described elsewhere (3). It is reduced to 5 individual sessions for one weekly session. It is administered by a physician or psychologist who has been trained in the stress management module and has not completed the initial assessment and inclusion of the patient. Nor will he have access to the results of the assessment scales. Each session lasts approximately 30-45 minutes (total 3-4H30 hours). It proposes a specific theme for each session according to the program described below. At the end of each session, the subject is asked to perform assigned tasks described in the protocol in order to acquire the therapeutic tools to better manage stress and anxiety (20 'at least 5 days out of 7). The total expected duration of the module's teaching is approximately 10 hours in total (3-4 hours in-class + 6-7 hours of training per task assigned outside the sessions).

Session 1 Information about stress, principes of techniques and program
Session 2 Techniques of relaxation
Session 3 Cognitive restructuring
Session 4 Mindfulness and acceptation
Session 5 Exposure and positives attitudes

Stress Management Program on digital support Group B

The program includes five 1-hour weekly sessions that patients follow from a website accessible on a computer in our unit.

Clinician-guidance Participants have minimal clinician assistance, called “minimal contact”, to encourage adherence and engagement with the program. This minimal contact of five minutes is performed before and after every session by a trained nurse, who investigates the adverse events and drug dose changes since the last session, answers any questions, discusses the progress of the session and possibly guides them in the navigation of the computer program.

Digital tools To avoid connection problems, a USB key is supplied to the patient at the first session and contains videos, audio files, self-help books, portfolios in the form of an e-guide, and a log book with the program of exercises to be completed between two successive sessions of the program. The patient is encouraged to practice one or several daily exercises with for twenty minutes each, five or six days per week.

In the Internet-based group, patients have minimal contact with any member from the medical staff before and after every session. In the first session, a USB key is supplied to the patient, containing videos, audio files, a self-help book portfolio in the form of an e-guide and log books providing the exercises to be completed between two sessions of the 5-session program. The patient is encouraged to practice a 20-minute daily exercise five or six times per wee.k

The program includes five 1-hour weekly sessions that patients follow from a computer in our unit.

A USB key is supplied to the patient from the first session, containing videos, audio files, self-help books, portfolio in the form of e-guide, and a log book with the program of the exercises to be realized between two successive sessions of the program. The patient is encouraged to practice one or several daily exercises with a duration of twenty minutes each, five or six days per week "

For group A and B

At the end of each session, the participants have a new contact time (5 minutes) with the same member of the research team to take stock of the session, answer any questions and schedule the tasks at home and to evaluate the occurrence of adverse reactions. A logbook will be given to the patient so that he can report the daily connection times and the daily exercise times. In the the case of the occurence of a problem, patients will be referred to their centre. In case of emergency, they can also be received by a resident in psychiatry of the referral hospital.

The 5 sessions of the two protocols will take place over a period of 4 consecutive weeks. In case of clinical aggravation, patients can be adressed for by the Stress and Anxiety Counseling Service at any time during the procedure, and if necessary, leave the protocol and after the procedure. Patients who would be absent exceptionally at one of the scheduled sessions could be received within 48 hours to complete their session. After 48 hours, and in the absence of 2 sessions or more, the patient will be excluded from the study.

Usual support Group C

Control group patients received usual care consisting of contact with their general practitioner and non-specific psychoeducation about stress and anxiety. Medication is prescribed as a stable dose during the period prior to assessment at 2 months. At 2 months, the patients of the Group C will benefit from a therapy according to the self-help program of their choice.

5.3.3 Assessment at 2 months

The assessment of group A, B and C will take place in consultation, 8 weeks after inclusion, by the self-questionnaires in paper version. A monitoring of care contacts, drug consumption, alcohol, tobacco, toxic, as well as any adverse events will be carried out. Estimated lenght: 50 minutes.

5.3.4 Assement at 6 months

Assessment at 6 months for the three groups (A, B and C) will be carried out in consultation in the same way as the 2 months assessment. In the event of a patient's inability to move to the centers, a letter containing the self-questionnaires will be sent to the patient.

5.3.5 Concurrent therapy

Concurrent therapies including psychotropic drugs are permitted during the study. Treatments initiated before inclusion will be maintained. No change in treatment will be made before the visit at 2 months.

5.3.6 Indemnity

Each participant included in the study will receive a lump sum indemnity of 45 euros in compensation for the travel expenses for each of the visits carried out as part of the studv

5.3.7 Flow-chart of the study

Tasks/Evaluations	Duration	**V0** Inclusion		**V1** (2 months)	**V2** (6 months)
Information and informed consent	15’	X	RANDOMIZATION in one the 3 groupe (A, B or C)		
ADA Diagnostic (DSM-5 criteria)	5’	X			
Inclusion and exclusion criteria	1’	X			
MINI	20’	X			
Urine Pregnancy test (women)	5’	X			
Sociodemographic data	5’	X			
Program (Face to face or digital support)					
**Self Reports**					
HADS	3’	X		X	X
VAS-stress	1’	X		X	X
Perceived Stress Scale	5’	X		X	X
Spielberger State-Trait Anxiety Inventory STAI	5’	X		X	X
Penn State Worry Questionnaire PSWQ	5’	X		X	X
Beck Depression Inventory BDI	5’	X		X	X
Global satisfaction	5’	X		X	X
Care contacts and medications	10’	X		X	X
5 weekly sessions	30’ à1h15 session			A B *	A B C*

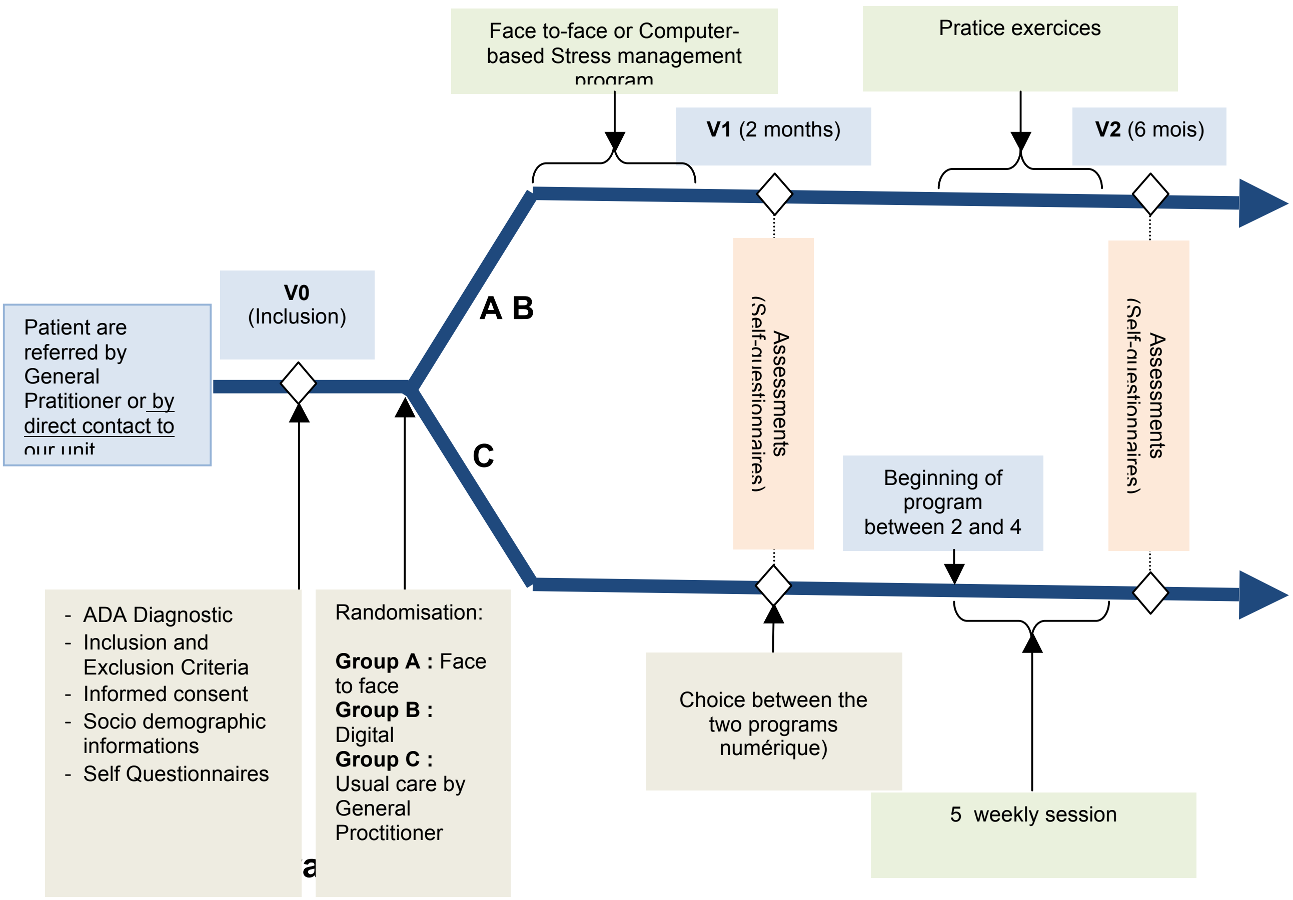

- Duration of inclusion period: 24 months
- Duration of participation in a study: 6 months
- Duration of data analysis: 6 months
- Duration of research: 3 years

5.5 Withdrawal from the study

The sponsor reserves the right to discontinue the study due to a lack of participants. The study may be terminated by decision of the competent joint authority, the sponsor or the coordinating clinical investigator.
The patient have the right to withdraw from the study at any time, for any reason.

5.6 Exclusion period

Participation of patients in other studies is not allowed until the visit at two monts. Exclusion period is not necessary because there are no invasive intervention and no drugs in this study.

5.7 Benefits and risks of the study

5.7.1 Benefits

The benefits of participating of this study for the patient suffering from ADA is : to learn self help stress managementand ; to reduce anxiety level ; to cope in a different way with stress in daily life.
Self help stress management program is recommended by the French National Authority for Health (HAS) to reduce benzodiazepine use in patients suffering from anxiety disorders.
All treatments are maintained (medical care and medication). This is a complementary measure beneficial to the patient.

5.7.2 Risks

None except an psychological impact during assessement by questionnaire.

5.8 Independent Monitoring Committee

Independant Safety Monitoring Commitee may not be needed :

No drug medication
No invasive intervention
Insignificant impact of the study
Expected Benefit for all the participants

VI Stress Management Program

6.1 Stress management program face to face with a therapist Group A

Course of the 5 sessions

Session 1 Understaning and recognizing stress

Content: What is stress? How modern everyday life can affect our stress levels and explanation of stress as a reaction to real or perceived threats to one's physical and mental well-being. Cognitive behavioral stress model (according to Lazarus' Model) The relationship between emotion, cognition and behavior illustrated by a practical exemple (when I am stuck in traffic' What I think about ? What I feel ? What I do ?)' . Identificate physiological (heart rate, dyspnea, checking…) and psychological stress symptoms (negative automatic thoughts, fear, anger…). Information about program structure.

Exercice in session: Identify the different type of stress, big stressors (eg, wedding, pregnancy, a new job) and everyday stressors or concerns (eg, noisy neighbors, parking ticket, a dreaded phone call). discuss treatment goals and expectations.

Homework: Identify everyday stressor. Hierarchy of the differnent stress from the minor to the most important. Describe the stress reaction.

Session 2 Physical relaxation

Content : Introducing different relaxation techniques : deep breathing, progressive muscle relaxation, visualisation and their relevance to stressful situations;

Guided Muscle Relaxation is derived from a traditional progressive muscle relaxation activity,

Exercice : In session experiential exercices
- Deep breathing : Breath slowly, abdominal breathing, breath in during 4 seconds and breath out during 6 seconds.
- Muscular relaxation : tense and release different muscular groups.

Mental relaxation : relax mind and body with deep breathe and pleasant visualisation

Homework : Deep breathing 5 minutes two or three times a day. Muscle relaxation or mental relaxation, depending on their own preference 10-15' Twice a day. Subsequently, they practised 6 times a day.

Session 3 Cognitive restructuring

Content : Cognitive basis of stress. The relationship between emotion, cognition and behavior. Recognizing automatic thoughts of their own. How to apply in to the real life with examples. Cognitive reappraisal (reconsidering the stressor from a different perspective). Identify thought pattern that can cause stress (such as all-or nothing thought, should be though, self-criticisme, over-interpreting) to help subjects become aware of their own though patterns. To introduce the concept of changing thoughts;

to explain common thinking-errors, alternative thoughts and coping statements; to practice it.

Exercice : Participants were taught to recognize events that trigger worrying and ruminating and challenging dysfunctional thoughts. Fill out the column worksheet to aid in the application of cognitive restructuring to real-life situations. Column including situation automatic thoughts emotion and feelings.

Homework : For homework participant use the column method. Cognitive work on automatic thoughts based on concrete situations in everyday life; rationalization of the content of thoughts through cognitive restructuring and identification of repetitive negative ruminations; daily exercises: identify dysfunctional automatic thoughts, search for other less stressful thoughts.

Session 4 Be aware to the present moment

Content : Introduction on Mindfulness. Mindful attitudes and nature of suffering. Interest of a short Mindfulness Based intervention. Ways to stay mindful to cope stress in daily life. Being in the present moment. Observing thoughts, feelings, and sensations as they are. Mindfulness is an effective intervention for stress. Mindfulness involves being in the present moment and accepting thoughts and feelings as they occur in a nonjudgmental way.

Exercise : To direct attention to breathing and bodily sensations, to overcome stressful thoughts, to accept the world and its surrounding thoughts;
Mindfulness breathing exercise to help become grounded in the present moment.
Body scan" exercise to increase bodily awareness of how one is doing and become more present in the moment.
Exercise to help become grounded in the present moment by focusing on an external sound. Negative (and positive) thoughts come and go, just stand by and watch your thoughts pass by.

Homework : exercise for relating to difficult or unpleasant situations, thoughts, or feelings. Focus attention on the present moment; to focus on sensations, to observe negative (or positive) thoughts and sensations in accepting rather than repressing emotions. Exercise of full consciousness by centration on the present moment and daily activity (walking, washing, eating…)

Session 5 Exposure and developing positive attitudes

Content: Principles of problem-solving, exposition, time management, planning pleasant activities, developing empathy, assertiveness.

Exercises : During the intervention participant build a hierarchy of stress-inducing situations and expose themselves to these stress condition gradually. The participant make an inventory of avoidance and safety behaviors. The participants learned how to set priorities, to reject requests that they found too demanding, to plan time for relaxation and self-reward, and to avoid unrealistic scheduling.

Homework : Increasign the number of pleasant activities assertivemess cognitive restructuring. The participant complete exposure exercise as homework assignments Engage in physical activity. Optimism and commitment lead to less stress

The participant is encouraged to practice alone all the stress management techniques and use it in the daily life.

Monitoring stress. Identifying everyday stressors and finding ways to deal with these. Strategies for dealing with everyday stressors: (1) avoidance—avoid situations that cause you stress or negative emotions; (2) change—if avoidance fails, adjust the situation such that the cause of stress dissipates; and (3) reattribute—if changing fails, think of the positives that come out of the stressful situation. Practice Deep breath , relaxation and mindfullness

6.2 Stress management program on digital support Group B

The self-help module is based on the same content and the same number of sessions (5 sessions for one weekly session), this time self-administered in the form of digital media available at each session *:*

- a presentation video
- a slideshow
- a chapter of self help guide downloadable in paper version
- audio exercises in the form of audio file (for sessions 2 and 4)
- an exercise book to practice during the home session downloadable in paper version

Participant were provided audio file and encouraged to practice

Seance 2 Exercises were provided and presented as an audio file

- I learn to breathe through the belly 5'01
- I slow down my breath 4'05
- I relax by breathing 6'58
- I release stress by breathing 2'52
- I breathe to install the trigger 2'55

Seance : 5 exercices were provided and presented as an audio file.

'I focus my attention on my breathing'
' I observe my body and my sensations'
'I observe my thoughts and I accept them'
' I accept the world and sound aroud me'
'I focus my attention on the present moment'

VII Adverse Events

7.1- Definitions

Adverse event: any untoward event occurring in a subject who is a participant in biomedical research regardless of whether the untoward event is related to the research or the research product.

Adverse reaction: any untoward effect related to research

Severe adverse events or reactions (SAE or SAR): any adverse event or effect that leads to:

- death
- is life threatening to subject participating in the research
- leads to hospitalization or requires longer than expected hospitalization
- leads to a long-lasting or severe handicap
- leads to a birth defect regardless of the administered dose
- any effect that is considered medically severe by the investigator
-

Unexpected adverse reaction: any adverse effect that differs from the information provided on the nature, the progression and the severity of effects expected by the products, applications and methodologies used during the study.

7.2 - Adverse effects and risks related to the protocol

None except an psychological effect of the passassion of the assements.

7.3 - Procedures to collect and report adverse effects

<u>Responsibilities of the investigator</u>

The investigator shall notify the sponsor immediately without delay when he/she becomes aware of any serious adverse events during the trial period.

All serious adverse events must be reported on the form "Serious Adverse Events" present in the case report file. This form must be sent to the sponsor (Notification Team of the Clinical Research Federation) by fax 03 20 44 57 11.

For each adverse event, the investigator assesses the severity and causal link between the adverse event and:

- the study medication,
- or the protocol.

Monitoring of adverse events will be provided by the investigator.

<u>Responsibilities of the sponsor</u>

<u>Reporting serious adverse events and adverse effects</u>

For each event or serious adverse event, the sponsor evaluates the severity and causal link between the event or adverse reaction:

- the study medication,
- or the protocol,

as well as the unexpected nature.

The sponsor will inform relevant Regulatory Authorities and the Ethics Committee:

- of all relevant information about serious unexpected adverse events suspected to be related to the study medication that are fatal or life threatening as soon as possible, and in any case no later than seven days after knowledge of such a case. Relevant follow-up information for these cases will subsequently be submitted within an additional eight days.
- of all other serious unexpected events suspected to be related to the study medication as soon as possible , but within a maximum of fifteen days of first knowledge by the investigator.

Statement to investigators

The sponsor shall inform all investigators working on the study about serious adverse events or adverse effects that could have an adverse impact upon the safety of persons who have consented to the research.

Annual Safety Report

Once a year for the duration of the study, or upon request, the sponsor transmits to the ANSM and CPP a safety report. This safety report shall include a comprehensive analysis of the safety profile of the study protocol taking into account all the relevant new safety data. The safety information will appear in tabular form summarising the serious adverse events or adverse effects in the biomedical research.

VIII Access to data sources

The investigators will provide access to data sources to members of the competent administrative authority for auditing, monitoring or inspection purposes.
Investigators agree to be audited and provide access to study data sources(medical records, computer files, study documents ...). During these visits subject to agreement by the investigator, the following may be audited:

- Compliance with the protocol and associated procedures
- Quality assurance of collected data in case reports: accuracy, missing data, data consistency, control of source documents.

IX Control and quality insurance

Trial schedule

A kick-off meeting to begin the study will be organized in each center with the Principal Investigator (PI) and a sponsor representative. The aim of kick-off meeting is to explain the protocol and trial schedule as well as the roles and responsibilities of the investigator and sponsors (CGP, monitoring, safety). The PI will keep the sponsor informed of the enrollment schedule.

Study monitoring

To ensure accurate data collection, case report forms (CRFs) will be checked regularly by the sponsor's Clinical Research Associate (CRA). During trial site monitoring visits CRAs will have free access to:

- CRFwith data from patients included in the study;
- medical files and nursing files for these patients;
- the investigator's notes.

This protocol has been assigned to the low risk research category. The goal of monitoring will therefore be to :

- confirm that patients are enrolled and have signed the informed consent form;
- confirm that inclusion criteria are respected;
- confirm the main primary end-point
- follow and report SAE
- identify any unexpected events that may require amendment.
- monitor the management of the pharmacy

Data collection

Data will be recorded in an electronic case report forms (eCRF), developed using Clinsight (ENNOV). Clinsight is certified for the management of clinical trials and meets the recommendations of the US Food and Drug Administration. The eCRF will be used to data entry in each investigator site and every center will be responsible of the patient's anonymization. The eCRF will be created, tested and validated before the start of data entry. The data necessary for monitoring the primary and secondary endpoints will be identified and managed at regular intervals throughout the trial. Data will be monitored by the data management team of the data-management department of University Hospital of Lille by using the predefined data management rules and queries will be automatically edited. Finally, an overall automated monitoring will be done by the data manager at the end of the data entry. In case of discrepancies, queries will be edited to resolve the problems encountered. After validation, the database will be frozen and exported to the statistical software package for analysis.

End of the study

At the end of the trial there will be a closing procedure. Documents and data will be classified. Once the final data analysis has been performed and validated, all of the data will be sealed and archived in a locked room.

La démarche Assurance Qualité qui sera mise en œuvre permet de prendre en charge les sujets se prêtant aux recherches dans les meilleures conditions de sécurité et de respect des règles médico-réglementaires.

X Ethical and legal consideration

The research will be performed in accordance with the protocol, good clinical practices, and the legislative and regulations in effect.

The investigator is responsible for the progression of the trial.

The investigator agrees:

- To keep data sources as well administrative documents in relation to the protocol,
- Not to include patients before receiving official approval from the Ethic's Committee and the expert authorities,
- To follow the protocol,
- To perform the trial in accordance with moral, regulatory, ethical and scientific principles governing clinical research,
- To obtained the informed, written consent from each participant,
- To report any serious adverse events.

The subjects will receive complete oral and written information explaining the steps of the trial. A letter of information will be given to the subject by the investigator or the doctor representing the investigator before the patient is included in the study.

An informed consent form (enclosed to the protocol) will be obtained from each patient before they are included in the study.

The informed consent form will be signed by the patient or if necessary another signatory may be added:

- A representative of the patient's guardian
- The family member or representative as defined by the Decree dated March 4, 2002 (for emergency consent or if it is impossible to obtain consent because of the patient's clinical condition).
- The witness (if it is impossible obtain written informed consent, verbal consent is obtained and witnessed)

No specific act of the protocol may begin without first obtaining the signed consent of the patient or his/her representative.

Three copies of the letter of information and the consent form will be drafted and 1 copy will be given to the patient or his/her representative.

The consent form will be signed by the investigator or the doctor representing the investigator and by the patient or his/her representative.

<u>Registration in the national register for individuals participating in biomedical research</u>

Any person that accepts to participate in the study will be registered by the investigator, in the national register of individuals participating in biomedical research.

Registration will be performed in accordance with decree dated November 14, 2006 in relation to gathering data in the national register of individuals participating in biomedical research.

<u>Approval from the Expert Authorities (EA) and the opinion of Ethics Committee (EC)</u>

The sponsor will submit a request for approval to the competent national authority (Agence Nationale de Sécurité des Médicaments, ANSM) and obtain approval form the Ethics Committee (Comité de Protection des Personnes, CPP) before beginning the study, in accordance with article L1121-4 of the Public Healthcare Code.

<u>Modifications to the protocol</u>

The sponsor is the only person that is authorized to modify the protocol after consulting with the coordinating investigator.

“Substantial modifications” mean modifications that significantly influence any aspect of the trial, in particular the protection of patients participating in the trial, including their safety, the validity of the study, the quality and safety of the tested products, the interpretation of the scientific documents explaining the phases of the study or its .

A request for a substantial modification shall be submitted by the sponsor either to the competent national authority or to the Ethics committee, or both, depending on the case, for approval and/or and opinion. As soon as the approval and/or positive opinion is/are received, an amended version of the protocol will be transmitted to the investigators by the sponsor.

A “non-substantial modification” means minor modifications or clarifications that do not affect the performance of the trial. These modifications are not submitted to the competent expert authorities but do require the agreement of the sponsor and the investigator and will be clearly documented (in the study file).

XI Data analyses and storage of research data documents

Personal data collected during the study will be reported on a laboratory notebook. After, it will be entered into a computerized data analysis system. Data will be confidential, in accordance with the law dated January 6, 1978.

Data will be analyzed in accordance with methodology described in MR 06001 of the CNIL in the plateform of epidemiology and biostatistics “Clinique de santé publique” of the CHU Lille managed by Prof Alain Duhamel.

Access to data will be restricted to individuals who are directly involved in the study. Data may be modified by any physician participating in the study or a fellow working with a physician participating in the study.

Trial data will be archived for at least 15 years after the trial has ended.

XII Study Funding and insurance

12.1 Funding

This project is sponsorised by The Clinical Research Hospital Program InterRégion of The French Ministry of Health est financé par le PHRCI.

12.2 Insurance

The sponsor has signed an insurance contract covering his liability and that of all the other participants in the study, in accordance with Article L1121-10 of the Code of Public Health.
Clinical trial participants will be provided on request with the conditions of insurance along with the patient information and consent form.

XIII Publication- Valorisation

The investigator study coordinator will prepare the final report of the study as required by law, and send to the developer.

In accordance with Article R 5121-13 of the Code of Public Health, the tests may not be subject to any written comments or oral without the joint agreement of the investigator and sponsor. Any publication must mention that the University Hospital of Lille applies as sponsor. In any event, the University Hospital of Lille, sponsor of the study, has the control of the first publication. The investigator shall send a copy of its publications to the proponent.

The sponsor is the exclusive owner of the results of the study. These results, as well as all data relating to the research, should never be given to third parties without previously negotiated by the Direction for Research in Health counterpart. Any such solicitation must be submitted as soon as possible for Legal Affairs of the Direction for Research in Health.

XIV Appendix

- References
- Diagnostic criteria of Adjustment disoerder with anxiety according DSM-5
- Mini Neuropsychiatric Interview MINI
- Hospital Anxiety and Depression Scale) (HADS) (French version)
- Penn State Worry Questionnaire PSWQ (French version)
- Spielberger State Anxiety Inventory (STAI) (French version)
- Visual Analogic Scale VAS (French version)
- Perceived Stress Scale PSS14 (French version)
- Beck Depression Inventory BDI (French version)

REFERENCES

1. Selye H. The stress concept in 1955. J Chronic Dis. 1955 Nov;2(5):583–92.
2. Selye H. The evolution of the stress concept: Stress and cardiovascular disease. Am J Cardiol. 1970 ;26(3):289–99.
3. Servant D. Gestion du stress et de l'anxiété. Paris: Elsevier Masson; 2012.
4. Servant D, Parquet PJ. Evenements de vie et anxiété. Encéphale 1994, 20 ; 333-7.
5. Lefebvre B, Poirot M. Stress et risques psychosociaux au travail: comprendre, prévenir, intervenir. Issy-les-Moulineaux: Elsevier Masson; 2011. 14-17 p.
6. Carta MG, Balestrieri M, Murru A et al. Adjustment Disorder: epidemiology, diagnosis and treatment. Clin Pract Epidemiol Ment Health CP EMH. 2009;5:15.
7. American Psychiatric Association, American Psychiatric Association. Diagnostic and statistical manual of mental disorders: DSM-5. 5th ed. Washington, D.C: American Psychiatric Association; 2013. 947 p.
8. Servant D, Pelissolo A, Chancharme L et al. Le trouble de l'adaptation avec anxiété. Caractéristiques cliniques et psychométriques chez des patients consultant en médecine générale. L'Encéphale. 2013 ;39 :347–51.
9. L'Agence nationale de sécurité du médicament et des produits de santé (ANSM). Etat des lieux en 2013 de la consommation des benzodiazépines en France [Internet]. 2013. Available from: http://ansm.sante.fr/S-informer/Points-d-information-Points-d-information/Etat-des-lieux-en-2013-de-la-consommation-des-benzodiazepines-en-France-Point-d-Information
10. ALD n°23. Troubles anxieux graves. Troubles anxieux graves. Haute Autorité de Santé (HAS) 2007.

11. Dugas MJ, Brillon P, Savard P et al. A Randomized Clinical Trial of Cognitive-Behavioral Therapy and Applied Relaxation for Adults With Generalized Anxiety DisorderBehav Ther. 2010 ; 41: 46–58
12. Leichsenring F, Salzer S, Jaeger U et al. Term Psychodynamic Psychotherapy and Cognitive-Behavioral Therapy in Generalized Anxiety
13. Ong L, Linden W, Young S. Stress management: What is it? J Psychosom Res. 2004 ;56 :133–7.
14. Turner L, Linden W, van der Wal R, Schamberger W. Stress management for patients with heart disease: a pilot study. Heart Lung J Crit Care. 1995 Apr;24(2):145–53.
15. Campbell TS, Stevenson A, Arena R, Hauer T, Bacon SL, Rouleau CR, et al. An investigation of the benefits of stress management within a cardiac rehabilitation population. J Cardiopulm Rehabil Prev. 2012 ;32:296–304.
16. Attari A, Sartippour M, Amini M et al. Effect of stress management training on glycemic control in patients with type 1 diabetes. Diabetes Res Clin Pract. 2006 ;73 :23–8.
17. Lopez C, Antoni M, Penedo F, Weiss D et al. A pilot study of cognitive behavioral stress management effects on stress, quality of life, and symptoms in persons with chronic fatigue syndrome. J Psychosom Res. 2011;70:328–34.
18. Antoni MH, Lechner S, Diaz A, Vargas S et al. Cognitive behavioral stress management effects on psychosocial and physiological adaptation in women undergoing treatment for breast cancer. Brain Behav Immun. 2009 ;23:580–91.
19. Stansfeld S, Candy B. Psychosocial work environment and mental health--a meta-analytic review. Scand J Work Environ Health. 2006 Dec;32(6):443–62.
20. Richardson KM, Rothstein HR. Effects of occupational stress management intervention programs: a meta-analysis. J Occup Health Psychol. 2008 Jan;13(1):69–93.
21. Cohen J A power primer. Psychological Bulletin 1992; 112 : 155-9.
22. Sharma V, Sood A, Prasad K et al. Bibliotherapy to decrease stress and anxiety and increase resilience and mindfulness: A pilot trial. Explore N Y N. 2014 ;10 :248–52.
23. Abramowitz JS, Moore EL, Braddock AE et al. Self-help cognitive-behavioral therapy with minimal therapist contact for social phobia: a controlled trial. J Behav Ther Exp Psychiatry. 2009 ;40:98–105.
24. Andersson G. Using the Internet to provide cognitive behaviour therapy. Behav Res Ther. 2009 ;47(3):175–80.
25. Matcham F, Rayner L, Hutton J, Monk A, Steel C, Hotopf M. Self-help interventions for symptoms of depression, anxiety and psychological distress in patients with physical illnesses: A systematic review and meta-analysis. Clin Psychol Rev. 2014 ;34 :141–57.
26. Kawai K, Yamazaki Y, Nakayama K. Process evaluation of a web-based stress management program to promote psychological well-being in a sample of white-collar workers in Japan. Ind Health. 2010;48:265–74.
27. Zetterqvist K, Maanmies J, Ström L et al. Randomized controlled trial of internet-based stress management. Cogn Behav Ther. 2003;32 :151–60.
28. Cook RF, Billings DW, Hersch RK, Back AS, Hendrickson A. A field test of a web-based workplace health promotion program to improve dietary practices, reduce stress, and increase physical activity: randomized controlled trial. J Med Internet Res. 2007;9(2):e17.
29. Beatty L, Lambert S. A systematic review of internet-based self-help therapeutic interventions to improve distress and disease-control among adults with chronic health conditions. Clin Psychol Rev. 2013 ;33 :609–22.
30. Rose RD, Buckey Jr. JC, Zbozinek TD et al. A randomized controlled trial of a self-guided, multimedia, stress management and resilience training program. Behav Res Ther. 2013 ;51 :106–12.
31. Heber E, Ebert DD, Lehr D, Cuijpers P, Berking M, Nobis S, Riper H. The Benefit of Web- and Computer-Based Interventions for Stress: A Systematic Review and Meta-Analysis. J Med Internet Res 2017 Feb 17;19(2):e32. PMID:28213341
32. Servant D, Rougegrez L, Barasino O, Demarty A-L, Duhamel A, Vaiva G. [Interest of computer-based cognitive behavioral stress management. Feasability of the Seren@ctif program]. Encephale 2016 Oct;42(5):415–420.
33. Sheehan DV, Lecrubier Y, Sheehan KH, Amorim P, Janavs J, Weiller E, Hergueta T, Baker R, Dunbar GC. The Mini-International Neuropsychiatric Interview (M.I.N.I.): the development and validation of a structured diagnostic psychiatric interview for DSM-IV and ICD-10. J Clin Psychiatry 1998;59 Suppl 20:22-33

34. Spielberger CD. Manual for the State-Trait Anxiety Inventory (Form Y) Self-evaluation questionnaire. Consult Psychol Press Palo Alto CA. 1983;
35. Olssøn I, Mykletun A, Dahl AA. The Hospital Anxiety and Depression Rating Scale: a cross-sectional study of psychometrics and case finding abilities in general practice. BMC Psychiatry. 2005;5:46.
36. Gosselin P, Dugas MJ, Ladouceur R et al. Evaluation of worry: validation of a French translation of the Penn State Worry Questionnaire. L'Encéphale. 2001;27:475–84.
37. Cohen S, Kamarck T, Mermelstein R. A global measure of perceived stress. J Health Soc Behav. 1983;24:385–96.
38. Lesage F-X, Chamoux A, Berjot S. Stabilité de l'échelle visuelle analogique dans l'évaluation du stress. Arch Mal Prof Environ. 2009 ;70:619–22.
39. Beck AT, Ward CH, Mendelson M et al. An inventory for measuring depression. Arch Gen Psychiatry. 1961 ; 4 : 561-571
40. Moher D, Schulz KF, Altman DG. The CONSORT statement: revised recommendations for improving the quality of reports of parallel-group randomised trials. Lancet 2001 Apr 14;357(9263):1191–1194.
41. Eysenbach G, CONSORT-EHEALTH Group. CONSORT-EHEALTH: improving and standardizing evaluation reports of Web-based and mobile health interventions. J Med Internet Res 2011 Dec 31;13(4):e126.
Disorder: A Randomized, Controlled Trial. Am J Psychiatry 2009; 166: 875–881
39 W. Seeman et al. Étude transversale de la prévalence du trouble de l'adaptation avec anxiété en médecine générale. L'Encéphale. 2001 juillet;27(3):238.

Adjustment Disorders Diagnostic Criteria DSM-5

A. The development of emotional or behavioral symptoms in response to an identifiable
stressor(s) occurring within 3 months of the onset of the stressor(s).

B. These symptoms or behaviors are clinically significant, as evidenced by one or both of
the following:
1. Marked distress that is out of proportion to the severity or intensity of the stressor, taking into account the external context and the cultural factors that might influence symptom severity and presentation.
2. Significant impairment in social, occupational, or other important areas of functioning.

C. The stress-related disturbance does not meet the criteria for another mental disorder
and is not merely an exacerbation of a preexisting mental disorder.

D. The symptoms do not represent normal bereavement.

E. Once the stressor or its consequences have terminated, the symptoms do not persist
for more than an additional 6 months.

Specify whether :
Acute, persistent (Chronic)

Specify whether:
309.0 (F43.21) With depressed mood: Low mood, tearfulness, or feelings of hopelessness are predominant.
309.24 (F43.22) With anxiety: Nervousness, worry, jitteriness, or separation anxiety is predominant.
309.28 (F43.23) With mixed anxiety and depressed mood: A combination of depression and anxiety is predominant.
309.3 (F43.24) With disturbance of conduct: Disturbance of conduct is predominant.
309.4 (F43.25) With mixed disturbance of emotions and conduct: Both emotional symptoms (e.g., depression, anxiety) and a disturbance of conduct are predominant.
309.9 (F43.20) Unspecified: For maladaptive reactions that are not classifiable as one of the specific subtypes of adjustment disorder.

American Psychiatric Association. (2013). Diagnostic and statistical manual of mental disorders: DSM-5. (5th ed.). Washington, D.C.: American Psychiatric Association. ISBN 0890425558.

Trouble de l'Adaptation avec Anxiété selon les critères du DSM-5

A. Développement de symptômes dans les registres émotionnels et comportementaux, en réaction à un ou plusieurs facteur(s) de stress identifiable(s), au cours des trois mois suivant la survenue de celui-ci (ceux-ci).
B. Ces symptômes ou comportements sont cliniquement significatifs, comme en témoignent :

(1) soit une souffrance marquée, plus importante qu'il n'était attendu en réaction à ce facteur de stress, en tenant compte du contexte externe et des facteurs culturels qui pourraient influencer la présentation et la sévérité des symptômes.

(2) soit une altération significative du fonctionnement social ou professionnel
C. La perturbation liée au stress ne répond pas aux critères d'un autre trouble spécifique de l'Axe I et n'est pas simplement l'exacerbation d'un trouble préexistant.
D. Les symptômes ne sont pas l'expression d'un Deuil.
E. Une fois que le facteur de stress (ou ses conséquences) a disparu, les symptômes ne persistent pas au-delà de 6 mois.

Aigu : si la perturbation persiste moins de 6 mois.
Chronique : si la perturbation persiste 6 mois ou plus. Par définition, les symptômes ne peuvent pas persister plus de 6 mois une fois que le facteur de stress ou ses conséquences ont disparu. Cette spécification s'applique donc lorsque la durée de la perturbation est plus importante que six mois, en réaction à un facteur de stress prolongé ou bien dont les conséquences sont durables.
Les Troubles de l'Adaptation sont codés par sous-types, qui sont sélectionnés en fonction des symptômes prédominants. Le facteur de stress (stresseur) spécifique peut être caractérisé sur l'Axe IV.
Les différents sous-types du TA sont les suivants : avec humeur dépressive, avec anxiété, avec à la fois anxiété et humeur dépressive, avec perturbation des conduites, avec perturbation à la fois des émotions et des conduites, non spécifié.
Le sous-type avec anxiété doit être utilisé lorsque les manifestations prédominantes sont des symptômes tels que nervosité, inquiétude ou agitation ou bien chez l'enfant, la peur de se séparer des personnes auxquelles il est le plus attaché.

Association Américaine de Psychiatrie. DSM-5. Manuel diagnostique et statistiques de trouble mentaux. 5ème édition. Elsevier Masson 2015, 1176 p. (Trad M-A Crocq, J-D Guelfi)

MINI : Mini International Neuropsychiatric Interview (version 5.0.0)
Y. Lecrubier, D. Sheehan et al., 1997

DIAGNOSTIC OF PSYCHIATRIC DISORDERS

	YES		NO	NK (not known)
	ACTUAL	PAST<br>If past disorder, identify the period		
MAJOR DEPRESSIVE EPISODE	* ☐ 15 days	☐	☐	☐
* Specify<br>*If melancholic features*	☐ 15 days		☐	☐
DYSTHYMIA	☐ 2 years		☐	☐
SUICIDAL IDEATION	☐ 1 month	☐	☐	☐
HYPOMANIAC EPISODE	☐ 1 month	☐	☐	☐
EPISODE MANIAQUE	☐ 1 month	☐	☐	☐
PANIC DISORDER without agoraphobia	☐ 1 month	☐	☐	☐
PANIC DISORDER with agoraphobia	☐ 1 month	☐	☐	☐
AGORAPHOBIA without Panic Disorder	☐ 1 month	☐	☐	☐
SOCIAL PHOBIA	☐ 1 month	☐	☐	☐
OBSESSIVE COMPULSIVE DISORDER	☐ 1 month	☐	☐	☐
POST TRAUMATIQUE STRESS DISORDER	☐ 1 month	☐	☐	☐
ALCOHOL DEPENDENCE	☐ 12 months	☐	☐	☐
ACOHOL ABUSE	☐ 12 months	☐	☐	☐
DRUG DEPENDENCE	☐ 12 months	☐	☐	☐
DRUG ABUSE	☐ 12 months	☐	☐	☐
PSYCHOTIC SYNDROME	☐ 1 month	☐	☐	☐
MOOD DISORDER<br>with psychotic features	☐ 1 month	☐	☐	☐
ANOREXIE MENTALE	☐ 3 months	☐	☐	☐
BOULIMIA	☐ 3 months	☐	☐	☐
ANOREXIA *Binge-eating / Purging type*	☐ 3 mois	☐	☐	☐
GENERALIZED ANXIETY DISORDER	☐ 6 months	☐	☐	☐
ANTISOCIAL PERSONNALITY DISORDER	☐ Life time		☐	☐

Echelle d’anxiété – dépression HADS

Lisez chaque question et entourez la réponse qui s'adapte le mieux à vous **pour la semaine passée**. Votre réponse ne doit pas être trop réfléchie mais rapide. Il n'y a pas de bonne ou de mauvaise réponse. N'entourez qu'une réponse par question.

---

1. Je me sens tendu ou énervé

- **0** Jamais
- **1** De temps en temps
- **2** Souvent
- **3** La plupart du temps

---

2. Je prends plaisir aux mêmes choses qu’autrefois

- **0** Oui, tout autant
- **1** Pas autant
- **2** Un peu seulement
- **3** Presque plus

---

3. J’ai une sensation de peur comme si quelque chose d’horrible allait m’arriver

- **0** Pas du tout
- **1** Un peu, mais cela ne m’inquiète pas
- **2** Oui, mais ce n’est pas trop grave
- **3** Oui, très nettement

---

4. Je ris facilement et vois le bon côté des choses

- **0** Autant que par le passé
- **1** Plus autant qu’avant
- **2** Vraiment moins qu’avant
- **3** Plus du tout

---

5. Je me fais du souci

- **0** Très occasionnellement
- **1** Occasionnellement
- **2** Assez souvent
- **3** Très souvent

---

6. Je suis de bonne humeur :

- **0** La plupart du temps
- **1** Assez souvent
- **2** Rarement
- **3** Jamais

---

7. Je peux rester tranquillement assis à ne rien faire et me sentir décontracté :

- **0** Oui, quoi qu’il arrive
- **1** Oui, en général
- **2** Rarement
- **3** Jamais

---

8. J’ai l’impression de fonctionner au ralenti :

- **0** Jamais
- **1** Parfois

**2**	Très souvent
**3**	Presque toujours

9. J'éprouve des sensations de peur et j'ai l'estomac noué :

**0**	Jamais
**1**	Parfois
**2**	Assez souvent
**3**	Très souvent

10. Je ne m'intéresse plus à mon apparence :

**0**	J'y prête autant d'attention que par le passé
**1**	Il se peut que je n'y fasse plus autant attention
**2**	Je n'y accorde pas autant d'attention que je le devrais
**3**	Plus du tout

11. J'ai la bougeotte et n'arrive pas à tenir en place

**0**	Pas du tout
**1**	Pas tellement
**2**	Un peu
**3**	Oui, c'est tout à fait le cas

12. Je me réjouis d'avance à l'idée de faire certaines choses :

**0**	Autant qu'avant
**1**	Un peu moins qu'avant
**2**	Bien moins qu'avant
**3**	Presque jamais

13. J'éprouve des sensations soudaines de panique :

**0**	Jamais
**1**	Pas très souvent
**2**	Assez souvent
**3**	Vraiment très souvent

14. Je peux prendre plaisir à un bon livre ou à une bonne émission radio ou de télévision :

**D**	**0**	Souvent
	**1**	Parfois
	**2**	Rarement
	**3**	Très rarement

Calculez le score de l'échelle HAD pour la dépression, correspondant au total des valeurs des questions paires ☐

Pour que le sujet soit inclus dans l'étude, le score doit être ≤ 10

Questionnaire sur les inquiétudes du Penn-State

Veuillez utiliser l'échelle ci-dessous pour exprimer jusqu'à quel point chacun des énoncés suivants **vous correspond actuellement** (écrivez le numéro vous représentant en tête de chacun des énoncés).

1	2	3	4	5
Pas du tout caractéristique	Un peu caractéristique	Assez caractéristique	Très caractéristique	Extrêmement caractéristique

	1.	Si je n'ai pas assez de temps pour tout faire, je ne m'en inquiète pas
	2.	Mes inquiétudes me submergent
	3.	Je n'ai pas tendance à m'inquiéter à propos des choses
	4.	Plusieurs situations m'amènent à m'inquiéter
	5.	Je sais que je ne devrais pas m'inquiéter, mais je n'y peux rien
	6.	Quand je suis sous pression, je m'inquiète beaucoup
	7.	Je m'inquiète continuellement à propos de tout
	8.	Il m'est facile de me débarrasser de pensées inquiétantes
	9.	Aussitôt que j'ai fini une tâche, je commence immédiatement à m'inquiéter au sujet de toutes les autres choses que j'ai encore à faire
	10.	Je ne m'inquiète jamais
	11.	Quand je ne peux plus rien faire au sujet d'un souci, je ne m'en inquiète plus
	12.	J'ai été un inquiet tout au long de ma vie
	13.	Je remarque que je m'inquiète pour certains sujets
	14.	Quand je commence à m'inquiéter, je ne peux plus m'arrêter
	15.	Je m'inquiète tout le temps
	16.	Je m'inquiète au sujet de mes projets jusqu'à ce qu'ils soient terminés

Inventaire d’anxiété Trait-Etat de Spielberger (STAI forme Y-A)

Un certain nombre de phrases que l'on utilise pour se décrire sont données ci-dessous. Lisez chaque phrase, puis marquez d'une croix, parmi les quatre points à droite, celui qui correspond le mieux à ce que vous ressentez **à l'instant, juste en ce moment**.
Il n'y a pas de bonnes ni de mauvaises réponses. Ne passez pas trop de temps sur l'une ou l'autre de ces propositions, et indiquez la réponse qui décrit le mieux vos sentiments actuels.

		Non	Plutôt non	Plutôt oui	Oui
1	Je me sens calme				
2	Je me sens en sécurité, sans inquiétude, en sûreté				
3	Je me sens tendu(e), crispé(e)				
4	Je me sens surmené(e)				
5	Je me sens tranquille, bien dans ma peau				
6	Je me sens ému(e), bouleversé(e), contrarié(e)				
7	L’idée de malheurs éventuels me tracasse en ce moment				
8	Je me sens content(e)				
9	Je me sens effrayé(e)				
10	Je me sens à mon aise				
11	Je sens que j’ai confiance en moi				
12	Je me sens nerveux (nerveuse), irritable				
13	J’ai la frousse, la trouille (j’ai peur)				
14	Je me sens indécis(e)				
15	Je suis décontracté(e), détendu(e)				
16	Je suis satisfait(e)				
17	Je suis inquiet, soucieux (inquiète, soucieuse)				
18	Je ne sais plus où j’en suis, je me sens déconcerté(e), dérouté(e)				
19	Je me sens solide, posé(e) pondéré(e), réfléchi(e)				
20	Je me sens de bonne humeur, aimable				

Echelles visuelles analogiques

Sur les échelles visuelles suivantes, indiquez par un trait vertical l'état où vous vous situez **actuellement** par rapport aux extrêmes.

Pas du tout stressé(e) — Extrêmement stressé(e)

Pas du tout satisfait — Extrêmement satisfait(e)

Echelle de stress perçu de COHEN - PSS14

Echelle de stress perçu
(d’après Cohen et Williamson 1988)

Au cours du dernier mois combien de fois	Jamais 1	Presque Jamais 2	Parfois 3	Assez souvent 4	Souvent 5
1 avez vous été dérangé (e) par un événement inattendu ?					
2 vous a - il semblé difficile de contrôler les choses importantes de votre vie ?					
3 vous êtes vous senti(e) nerveux (nerveuse) et stressé (e) ?					
4 avez vous affronté avec succès les petits problèmes et ennuis quotidiens,					
5 avez vous senti que vous faisiez face efficacement aux changements importants qui survenaient dans votre vie ?					
6 vous vous êtes senti(e) confiant dans vos capacité à prendre en main vos problèmes personnels,					
7 avez-vous senti que les choses allaient comme vous le vouliez					
8 avez-vous pensé que vous ne pouviez pas assumer toutes les choses que vous deviez faire ?					
9 avez vous été capable de maîtriser votre énervement ?					
10 avez vous senti(e) que vous dominiez la situation ?					
11 vous êtes vous senti(e) irrité(e) parce que les événements échappaient à votre contrôle ?					
12 vous êtes-vous surpris à penser à des choses que vous deviez mener à bien ?					
13 avez-vous été capable de contrôler la façon dont vous passiez votre temps ?					
14 avez vous trouvé que les difficultés s’accumulaient à un tel point que vous ne pouviez les contrôler ?					

Beck Depression Inventory BDI abrégé

Ce questionnaire comporte plusieurs séries de quatre propositions.

Pour chaque série, lisez les quatre propositions, puis choisissez celle qui décrit le mieux **votre état actuel**.

Cochez le numéro qui correspond à la proposition choisie. Si, dans une série, plusieurs propositions paraissent convenir, entourez les numéros correspondants.

**A.**	**Je ne me sens pas triste.**	**0** ☐
	Je me sens cafardeux ou triste.	**1** ☐
	Je me sens tout le temps cafardeux ou triste, et je n'arrive pas à en sortir.	**2** ☐
	Je suis si triste et si malheureux que je ne peux pas le supporter.	**3** ☐
**B.**	**Je ne suis pas particulièrement découragé ni pessimiste au sujet de l'avenir.**	**0** ☐
	J'ai un sentiment de découragement au sujet de l'avenir	**1** ☐
	Pour mon avenir, je n'ai aucun motif d'espérer.	**2** ☐
	Je sens qu'il n'y a aucun espoir pour mon avenir, et que la situation ne peut s'améliorer.	**3** ☐
**C.**	**Je n'ai aucun sentiment d'échec de ma vie.**	**0** ☐
	J'ai l'impression que j'ai échoué dans ma vie plus que la plupart des gens.	**1** ☐
	Quand je regarde ma vie passée, tout ce que j'y découvre n'est qu'échecs.	**2** ☐
	J'ai un sentiment d'échec complet dans toute ma vie personnelle (dans mes relations avec mes parents, mon mari, ma femme, mes enfants).	**3** ☐
**D.**	**Je ne me sens pas particulièrement insatisfait.**	**0** ☐
	Je ne sais pas profiter agréablement des circonstances.	**1** ☐
	Je ne tire plus aucune satisfaction de quoi que ce soit.	**2** ☐
	Je suis mécontent de tout	**3** ☐
**E.**	**Je ne me sens pas coupable.**	**0** ☐
	Je me sens mauvais ou indigne une bonne partie du temps.	**1** ☐
	Je me sens coupable.	**2** ☐
	Je me juge très mauvais et j'ai l'impression que je ne vaux rien.	**3** ☐
**F.**	**Je ne suis pas déçu par moi-même.**	**0** ☐
	Je suis déçu par moi-même.	**1** ☐
	Je me dégoûte moi-même.	**2** ☐
	Je me hais.	**3** ☐

**G.**	**Je ne pense pas à me faire du mal.**	**0** ☐
	Je pense que la mort me libérerait	**1** ☐
	J'ai des plans précis pour me suicider	**2** ☐
	Si je le pouvais je me tuerais	**3** ☐

**H.**	**Je n'ai pas perdu l'intérêt pour Ies autres.**	**0** ☐
	Maintenant, je m'intéresse moins aux autres gens qu'autrefois.	**1** ☐
	J'ai perdu tout l'intérêt que je portais aux autres gens, et j'ai peu de sentiments pour eux.	**2** ☐
	J'ai perdu tout intérêt pour les autres, et ils m'indiffèrent totalement.	**3** ☐

**I.**	**Je suis capable de me décider aussi facilement que de coutume.**	**0** ☐
	J'essaie de ne pas avoir à prendre de décision.	**1** ☐
	J'ai de grandes difficultés à prendre des décisions.	**2** ☐
	Je ne suis plus capable de prendre la moindre décision.	**3** ☐

**J.**	**Je n'ai pas le sentiment d'être plus laid qu'avant.**	**0** ☐
	J'ai peur de paraître vieux ou disgracieux.	**1** ☐
	J'ai l'impression qu'il y a un changement permanent dans mon apparence physique qui me fait paraître disgracieux.	**2** ☐
	J'ai l'impression d'être laid et repoussant.	**3** ☐

**K.**	**Je travaille aussi facilement qu'auparavant.**	**0** ☐
	Il me faut faire un effort supplémentaire pour commencer à faire quelque chose.	**1** ☐
	Il faut que je fasse un très grand effort pour faire quoi que ce soit.	**2** ☐
	Je suis incapable de faire le moindre travail.	**3** ☐

**L.**	**Je ne suis pas plus fatigué que d'habitude.**	**0** ☐
	Je suis fatigué plus facilement que d'habitude	**1** ☐
	Faire quoi que ce soit me fatigue.	**2** ☐
	Je suis incapable de faire le moindre travail.	**3** ☐

**M.**	**Mon appétit est toujours aussi bon.**	**0** ☐
	Mon appétit n'est pas aussi bon que d'habitude.	**1** ☐
	Mon appétit est beaucoup moins bon maintenant.	**2** ☐
	Je n'ai plus du tout d'appétit.	**3** ☐
